# Magnetic-Fe/Fe_3_O_4_-nanoparticle-bound SN38 as carboxylesterase-cleavable prodrug for the delivery to tumors within monocytes/macrophages

**DOI:** 10.3762/bjnano.3.51

**Published:** 2012-06-13

**Authors:** Hongwang Wang, Tej B Shrestha, Matthew T Basel, Raj Kumar Dani, Gwi-Moon Seo, Sivasai Balivada, Marla M Pyle, Heidy Prock, Olga B Koper, Prem S Thapa, David Moore, Ping Li, Viktor Chikan, Deryl L Troyer, Stefan H Bossmann

**Affiliations:** 1 Kansas State University, Department of Chemistry, CBC 201, Manhattan, KS 66506; 2 Kansas State University, Anatomy & Physiology, Coles 228, Manhattan, KS 66506; 3 Battelle Memorial Institute, 505 King Ave., Columbus, OH 43201; 4 University of Kansas, KU Microscopy & Analytical Imaging Laboratory, 1043 Haworth, Lawrence, KS 66045

**Keywords:** cell-based delivery, chemotherapeutic prodrug, magnetic Fe/Fe_3_O_4_ nanoparticles, SN38

## Abstract

The targeted delivery of therapeutics to the tumor site is highly desirable in cancer treatment, because it is capable of minimizing collateral damage. Herein, we report the synthesis of a nanoplatform, which is composed of a 15 ± 1 nm diameter core/shell Fe/Fe_3_O_4_ magnetic nanoparticles (MNPs) and the topoisomerase I blocker SN38 bound to the surface of the MNPs via a carboxylesterase cleavable linker. This nanoplatform demonstrated high heating ability (SAR = 522 ± 40 W/g) in an AC-magnetic field. For the purpose of targeted delivery, this nanoplatform was loaded into tumor-homing double-stable RAW264.7 cells (mouse monocyte/macrophage-like cells (Mo/Ma)), which have been engineered to express intracellular carboxylesterase (InCE) upon addition of doxycycline by a Tet-On Advanced system. The nanoplatform was taken up efficiently by these tumor-homing cells. They showed low toxicity even at high nanoplatform concentration. SN38 was released successfully by switching on the Tet-On Advanced system. We have demonstrated that this nanoplatform can be potentially used for thermochemotherapy. We will be able to achieve the following goals: (1) Specifically deliver the SN38 prodrug and magnetic nanoparticles to the cancer site as the payload of tumor-homing double-stable RAW264.7 cells; (2) Release of chemotherapeutic SN38 at the cancer site by means of the self-containing Tet-On Advanced system; (3) Provide localized magnetic hyperthermia to enhance the cancer treatment, both by killing cancer cells through magnetic heating and by activating the immune system.

## Introduction

Irinotecan (CPT-11) is a potent chemotherapeutic prodrug against various types of cancer, such as colorectal, lung, and ovarian cancer [1–5]. It is converted by carboxylesterase (predominantly in the liver) to its biologically active metabolite SN38 (7-ethyl-10-hydroxycamptothecin) [6–9]. Although CPT-11 had been approved as an anticancer agent by the US Food and Drug Administration (FDA) in 1997, the use of this prodrug is limited due to the low conversion rate (only 2–8%) of the administered dose into active SN38 in patients [10–11]. In addition, the conversion of CPT-11 into SN38 shows high interpatient variability because of the genetically different activity of carboxylesterase among individual patients [12–13]. Moreover, severe side effects, such as life-threatening diarrhea and neutropenia, have been observed [14–15]. SN38 is a topoisomerase I inhibitor, and it has demonstrated 100- to 1000-fold more cytotoxicity against various cancer cells in vitro than CPT-11 [6]. Despite the excellent anticancer potential, SN38 has not been used as an anticancer drug directly in humans due to its inherent poor solubility in any pharmaceutically acceptable media (solubility in water <5 µg/mL). To overcome this disadvantage of SN38, two major basic strategies have been developed. The first strategy is to directly introduce biocompatible hydrophilic functional groups to SN38 through chemical modification. A 40 kDa polyethylene glycol has been linked to the SN38 [16]. The highly water-soluble PEGylated SN38 (EZN-2208) demonstrated both drastic enhancement of its circulating half-life and preferential accumulation in solid tumors [17–19]. SN38 conjugated to a cationic peptide (Vectocell) by an esterase cleavable linker has been reported. The conjugate (DTS-108) is highly soluble in water and liberated significantly higher levels of free SN38 than CPT-11 did in a dog model [20]. An alternate strategy is to use delivery vehicles that can incorporate SN38 by chemical conjugation or physical entrapment. Polymeric micelles, liposomes and thermally sensitive polymer-based nanoparticles, as well as multi-armed-PEG-functionalized nanographene oxide, have been used as carriers for the delivery of SN38 into biological systems [21–26]. SN38-loaded polymeric micelles (NK012) have been used in preclinical and clinical studies against various types of cancer. Specific accumulation of this formulation to the tumor site by the EPR effect (enhanced permeation and retention), and sustained release of SN38 in tumor tissue have been observed [22]. Liposome encapsulation of SN38 (LE-SN38) enhances the solubility of SN38 and provides protection from rapid drug degradation. Increased cytotoxicity against various tumor cell lines and better therapeutic efficacy in xenograft mouse models, as compared to CPT-11, have been reported. Recently, we described a self-contained enzyme-activating prodrug cytotherapy for preclinical melanoma [27]. CPT-11 was loaded into double-stable RAW264.7 monocyte/macrophage-like cells (Mo/Ma) containing a Tet-On Advanced system for intracellular carboxylesterase (InCE) expression. The double-stable Mo/Ma homed to the lung melanoma within one day and successfully delivered the prodrug-activating enzyme/prodrug package to the tumors. Significantly reduced tumor weights and numbers were observed after activation of InCE. We also showed that these cells can carry the SN38–dextran irinotecan-like prodrug to the tumor site, and, upon activation of a previously silenced gene with doxycycline, significantly increased survival in a murine pancreatic cancer model in mice was observed [28]. Hyperthermia uses heat to kill cancer cells [29]. Numerous clinical trials have demonstrated that the combination of hyperthermia with radiation therapy and chemotherapy can greatly improve the efficacy of cancer treatment [30–31]. Ultrasmall magnetic nanoparticles generate heat efficiently in an alternating magnetic field (AMF). Due to their superior properties, such as negligible or low toxicity, biocompatibility, and potential for targeted accumulation at the tumor site, ultra-small magnetic nanoparticles are the prime candidates for application in magnetic hyperthermia [32–34]. We have developed a magnetic core/shell Fe/Fe_3_O_4_ nanoparticle platform, which can generate substantial heat within a magnetic field with low strength and frequency. Attenuation of mouse melanomas after AMF treatment was observed with both ligand-directed and cell-based cancer-specific delivery of magnetic nanoparticles [35–36]. When the nanoparticles were transported by RAW264.7 cells (monocyte/macrophage like cells) to the tumor site, survival of black mice bearing metastatic pancreatic tumors was increased by 31% after AMF treatment, compared to a nontreated control group [37].

In this report, we describe the synthesis of a prodrug combining SN38 and stealth core/shell Fe/Fe_3_O_4_ magnetic nanoparticles. The nanoparticles are functionalized with dopamine–oligoethylene glycol ligands, which make the nanoparticles both water-soluble and biocompatible. SN38 is covalently bound to the “tip” of the ligands by means of a carboxylesterase-cleavable linker. The nanoplatform can be loaded into double-stable RAW264.7 monocyte/macrophage-like cells (Mo/Ma) containing a Tet-On Advanced system for intracellular carboxylesterase (InCE) expression. Upon addition of doxycycline, SN38 is released from the nanoparticles, as evidenced by HPLC analysis. The nanoplatform shows efficient heating ability in an alternating magnetic field. This system can be potentially used as a multipurpose anticancer reagent for triggered thermochemotherapy. Delivery within Mo/Ma cells is capable of evading the reticuloendothelial system. Mo/Ma cells are known to integrate with the tumor tissue [28]. The activation of SN38 by InCE expression can be precisely timed. Localized hyperthermia has the potential to work in synergy with chemotherapy, especially because both hyperthermia and the activation of SN38 can be precisely and independently timed. Furthermore, hyperthermia is known to activate the immune system if the correct temperature is chosen [29].

## Experimental

### Materials

SN38 was purchased from Qventas (Newark, DE). Dopamine hydrochloride, Boc anhydride, benzyl bromide, trifluoroacetic acid, succinic acid anhydride, tetraethylene glycol, 4-piperidinecarboxylic acid, EDC, DMAP, CDI, Fe(CO)_5_, oleylamine, ODE, hexadecylamine, fetal bovine serum (FBS), neocuproine, ascorbic acid, ammonium acetate, and concentrated hydrochloric acid (HCl) were purchased from Sigma-Aldrich (St. Louis, MO). RAW264.7 mouse monocyte/macrophage (Mo/Ma) cells were purchased from ATCC (Manassas, VA). RPMI, Geneticin (G418), hygromycin and penicillin-streptomycin were purchased from Invitrogen (Carlsbad, CA). Thiazolyl blue and sodium dodecyl sulfate were purchased from Fisher Scientific (Pittsburgh, PA). Ferrozine was purchased from Hach (Loveland, CO).

#### Synthesis of core/shell Fe/Fe_3_O_4_ magnetic nanoparticles (MNPs)

**Scheme 1:**
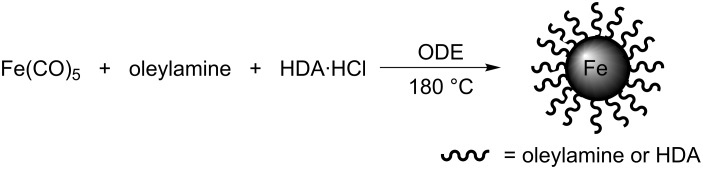
Preparation of core/shell Fe/Fe_3_O_4_ magnetic nanoparticles (MNPs).

The core/shell Fe/Fe_3_O_4_ magnetic nanoparticles were synthesized by extensive modification of a literature procedure originally described by Lacroix et al. [38] (Scheme 1). Thermal decomposition of iron pentacarbonyl (Fe(CO)_5_) in octadecene (ODE) under argon in the presence of oleylamine and hexadecylammonium chloride (HAD·HCl) at 180 °C gave highly crystalline iron(0) nanoparticles. When these nanoparticles were exposed to air at room temperature, a thin layer of Fe_3_O_4_ formed due to the oxidation of the nanoparticle surface, thus, core/shell Fe/Fe_3_O_4_ nanoparticles were constructed. The introduction of the Fe_3_O_4_ shell provides easy surface functionalization of the core/shell Fe/Fe_3_O_4_ nanoparticles. The obtained nanoparticles were washed with hexane and ethanol, collected by centrifugation, and dried under high vacuum for further use in this study.

#### Synthesis of a hydrophilic dopamine-anchored InCE-cleavable linker between SN38 and Fe/Fe_3_O_4_ magnetic nanoparticles

**Scheme 2:**
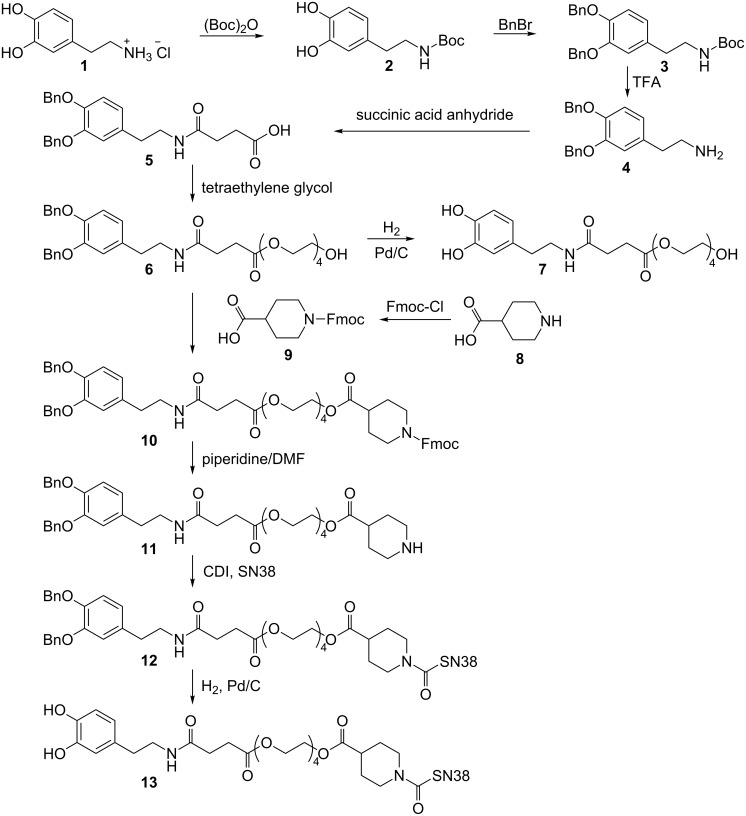
Functionalization of SN38.

The synthesis of a hydrophilic dopamine-anchored InCE-cleavable linker between SN38 and Fe/Fe_3_O_4_ nanoparticles was achieved in a 10-step reaction procedure, as described in Scheme 2. Briefly, after selective protection of the hydroxyl groups of dopamine **1** with benzyl bromide, the free amine group was reacted with succinic acid anhydride to form compound **5**. Tetraethylene glycol reacted with compound **5** in an EDC-coupling reaction to give compound **6**. Deprotection of the hydroxyl groups by Pd/C-catalyzed hydrogenation yielded compound **7**, which was used as ligand **I** to enhance the water solubility of the magnetic nanoparticles (MNPs). A piperidine moiety was introduced by reacting compound **6** with compound **9** to afford compound **10**. After removing the Fmoc group, compound **11** reacted with 10-OH of SN38 in the presence of CDI to give compound **12**. The final product **13** was obtained after deprotection of the hydroxyl groups by Pd/C-catalyzed hydrogenation, and was used as ligand **II** to incorporate SN38 to the MNPs. Compound **13** was fully characterized with ^1^H NMR, ^13^C NMR, and mass spectrometry.

#### Loading SN38 to Fe/Fe_3_O_4_ magnetic nanoparticles (MNPs)

Loading of SN38 to core/shell Fe/Fe_3_O_4_ magnetic nanoparticles was achieved by ligand exchange (chemisorptions) due to the much higher affinity of dopamine for the Fe_3_O_4_ surface compared to oleylamine [39–45]. A solution of compound **7** and compound **13** in DMF with a molar ratio of 10/1 was added to a dispersion of freshly synthesized Fe/Fe_3_O_4_ nanoparticles in hexane. After sonication for 5 min all the nanoparticles precipitated out, and the supernatant became a clear solution. After decanting of the supernatant, the nanoparticles were washed with hexane, DMF and ethanol to remove the free ligands. The obtained nanoparticles were dried in high vacuum.

#### Characterization of the Fe/Fe_3_O_4_ magnetic nanoparticles and the organic ligands

The morphology of the core/shell Fe/Fe_3_O_4_ magnetic nanoparticles loaded with or without SN38 was characterized by transmission electron microscopy (TEM). The TEM samples were prepared by immersing carbon-coated 200-mesh copper grids into a solution of drug-free or SN38-loaded MNPs followed by washing of the grids with dropwise chloroform and drying overnight in a desiccator. The dried grids were analyzed with a Philips CM100 microscope operated at 100 kV. High-resolution TEM was recorded on FEI Tecnai F20XT, 200 kV; FEI, Hilsboro, OR. Powder X-ray diffraction (XRD) patterns were obtained on a Bruker D8 X-ray diffractometer with Cu Kα radiation. The hydrodynamic diameter and the zeta potential of the MNPs were measured on a ZetaPALS zeta potential analyzer (Brookhaven Instruments Corporation) by hydrodynamic light scattering and laser Doppler electrophoresis. The ^1^H NMR and ^13^C NMR were obtained on a Varian Unity Plus (400 MHz) NMR spectrometer with deuterated chloroform or DMSO as solvents and TMS as the internal standard. ESI–MS spectra were acquired on an API4000 (Applied Biosystems, Foster City, CA) triple quadrupole mass spectrometer with electrospray ionization (ESI). Fluorescence measurements of free SN38 and SN38-loaded MNPs were performed on a Fluoro Max-2 instrument (HORIBA Jobin Yvon Company). The samples were excited at λ = 380 nm. UV–vis absorption analysis was carried out on a Cary 500 UV–vis–NIR spectrophotometer. The SN38 loading on the magnetic nanoparticles was determined by HPLC. The HPLC system consists of a Waters 1525 binary HPLC pump, Waters 1500 column heater, and Waters 2998 photodiode array detector. The Freeze 2 chromatographic software was used for data acquisition and processing. The quantification of SN38 was achieved on an Agilent Eclipse XDB-C8 (4.6 × 150 mm, 5 μm) analytical column by using a mobile phase consisting of a water and methanol gradient from 60/40 to 5/95 in 20 min with a total flow of 1 mL/min. SN38 was detected at an UV wavelength of 380 nm, and quantitatively determined by an internal calibration method with anthracene as the internal standard.

#### Alternating magnetic field (AMF) heating of the MNPs

For the measurement of the heating effect, an induction heater (Superior Induction Company, Pasadena, CA) was used. The heater contains a copper coil, one inch in diameter with four turns, and is continuously cooled with cold water. The heater was operated with 5 kA/m field amplitude and 366 kHz frequency. SN38-loaded Fe/Fe_3_O_4_ nanoparticles (MNP-SN38) were dispersed in water, and were subjected to the alternating magnetic field for 5 min. To measure the temperature change, a fiber optic probe (Neoptix, Quebec, Canada) was used.

#### Cell culture

RAW264.7 cells (mouse monocyte/macrophage-like cells, Mo/Ma) were cloned with the rabbit carboxylesterase (InCE) gene with Tet-On system and made double stable. Generation of the double-stable cells inducible for InCE (double-stable Mo/Ma) was described in an earlier paper [27]. Double-stable Mo/Ma were cultured in the RPMI medium containing 10% fetal bovine serum (Sigma) in a 37 °C humidified incubator with 5% CO_2_, with 100 µg/mL Geneticin (G418) and 100 µg/mL hygromycin added to preserve stable transfection.

#### Loading Mo/Ma with nanoparticles and determination of iron loading in Mo/Ma cells

To determine the loading of nanoparticles, Mo/Ma were plated in a six-well plate at a density of 300,000 cm^−2^, and incubated overnight at 37 °C to become 70% confluent. The next day, the medium was removed and 0 to 320 µg/mL of SN38-loaded Fe/Fe_3_O_4_ nanoparticles in fresh medium was added. After 24 h, the medium was removed; the cells were washed with 1× PBS three times, and stained with Prussian blue and counter stained by nuclear fast red to confirm that the loaded nanoparticles were iron/iron oxide nanoparticles.

#### Flow cytometry

Flow cytometry was used to determine the percentage of cells loaded with MNP. The cells were plated in six-well plates at a density of 300,000 cm^−2^ and allowed to attach overnight. The next day, the cells reached 70% confluence. They were then incubated with 0, 20, 40, 80, 160, 320 µg/mL of SN38-loaded Fe/Fe_3_O_4_ nanoparticles in fresh medium and incubated overnight. After taking up the nanoparticles, the cells were washed three times with 1× PBS and lifted by scraping. MNP loaded cells were analyzed by flow cytometry. Side scatter was used to determine the loading of the nanoparticles in the cells and compared to the side scatter of control cells. 10,000 cells were counted and analyzed. This procedure was repeated three times. Data were analyzed by using Cytosoft software (Guava Easycyte Plus System, Millipore Corporation, MA).

#### MTT Assay

The MTT assay [46] was carried out to determine the toxicity of NMP-SN38 on Mo/Ma. 3-(4,5-Dimethylthiazol-2-yl)-2,5-diphenyltetrazolium bromide (MTT) was dissolved in PBS at 5 mg/mL to prepare the MTT reagent solution. MTT solubilization buffer was prepared by dissolving 10% (w/v) sodium dodecylsulfate and 0.10 M HCl in water. To assay the cell viability, MTT reagent solution 1:10 (v/v, reagent solution/cell medium) was added to the cells and incubated at 37 °C for 4 h. After incubation, the MTT solution in buffer (1:1, medium/buffer) was added to the medium, incubated overnight, and the absorbance at 550 nm and 690 nm, as background absorbance, was measured by using a plate reader (spectraMAX 190, Molecular Device, Sunnyvale, California).

#### Ferrozine Assay

The iron content of the nanoparticles and the nanoparticle-loaded cells was determined by using the ferrozine assay [47]. SN38-loaded Fe/Fe_3_O_4_ nanoparticles (MNP-SN38) were loaded into double-stable Mo/Ma with 0, 20, 40, 80, 160, 320 µg/mL, as described before. The incubation time was 24 h. Then the cells were lifted, suspended in 2.0 mL of distilled water and lysed by using sonifire (sonicator) for 30 sec. Cell debris was removed by centrifugation at 1000 RPM for 3 min. MNP-SN38 were also suspended in 2 mL of distilled water for comparison purposes. HCl (0.5 mL; 1.5 M) and 0.20 mL of ascorbic acid (2.0 M) were added to each sample and incubated at 70 °C for 1 h. The ferrozine reagent solution was prepared as follows: 6.5 mM ferrozine, 13.1 mM neocuproine, 2.0 mM ascorbic acid and 5.0 M ammonium acetate in distilled water. After the incubation period, 0.20 mL of ferrozine reagent solution was added. The complexation of iron(II) was complete within 30 min at room temperature, as indicated by UV–vis absorption spectrometry. The absorbance was recorded at 562 nm. Iron solutions at 0, 0.1, 0.2, 0.5, 1.0, 2.0, and 5.0 µg/mL were prepared in distilled water by using 0.125 N ferrous ammonium sulfate. The ferrozine assay was then used to obtain a standard curve and to determine the iron content in the Fe/Fe_3_O_4_ nanoparticles.

## Results and Discussion

### Introducing hydrophilic dopamine to SN38

Dopamine has been reported as a robust anchor to immobilize functional groups on the surface of iron oxide nanoparticles [39–45]. Introducing polyethylene glycol to the dopamine anchor can greatly improve both the solubility and biocompatibility of iron oxide nanoparticles [48–49]. We have demonstrated in our previous papers that dopamine linked with simple tetraethylene glycol could sufficiently enhance the solubility and biocompatibility of core/shell Fe/Fe_3_O_4_ nanoparticles [35,37]. Here, we have conjugated the anticancer agent SN38 to the dopamine–tetraethylene glycol moiety via a carboxylesterase-cleavable linker, in analogy to the biochemical activation of CPT-11 [11]. We expect that through this design, two goals can be achieved:

(1) The hydrophilic dopamine functionalized SN38 prodrug is less toxic than SN38 itself, because structurally it is more like CPT-11.

(2) The SN38-prodrug can be immobilized on the water-soluble core/shell Fe/Fe_3_O_4_ magnetic nanoparticles through the dopamine anchor, and then the whole nanoplatform is loaded on double-stable monocyte/macrophage-like cells to specifically target the tumor, and release SN38 at the tumor site by the Tet-On Advanced system.

It is noteworthy that the hydrophilicity of the SN38 prodrug (log *P*(**13**) = 0.55) is higher than that for the tetraethylene glycol stealth ligand (log *P*(**7**) = −0.51), which is the reason why we have selected the molar ratio of 1/10 for **13** and **7** at the surface of the Fe/Fe_3_O_4_ nanoparticles. The pharmacologically active lactone form of SN38 is distinctly less hydrophilic (log *P* = 0.89). It can associate with cell membranes and diffuse into cells. Therefore it is very important that SN38 will be activated “on site” to minimize collateral damage. The construction of the nanoparticle-binding SN38 prodrug was achieved in a 10-stage synthesis with overall 32% yield. The final product was fully characterized by ^1^H NMR, ^13^C NMR, and mass spectrometry.

### Characterization of the nanoparticles

**Figure 1:**
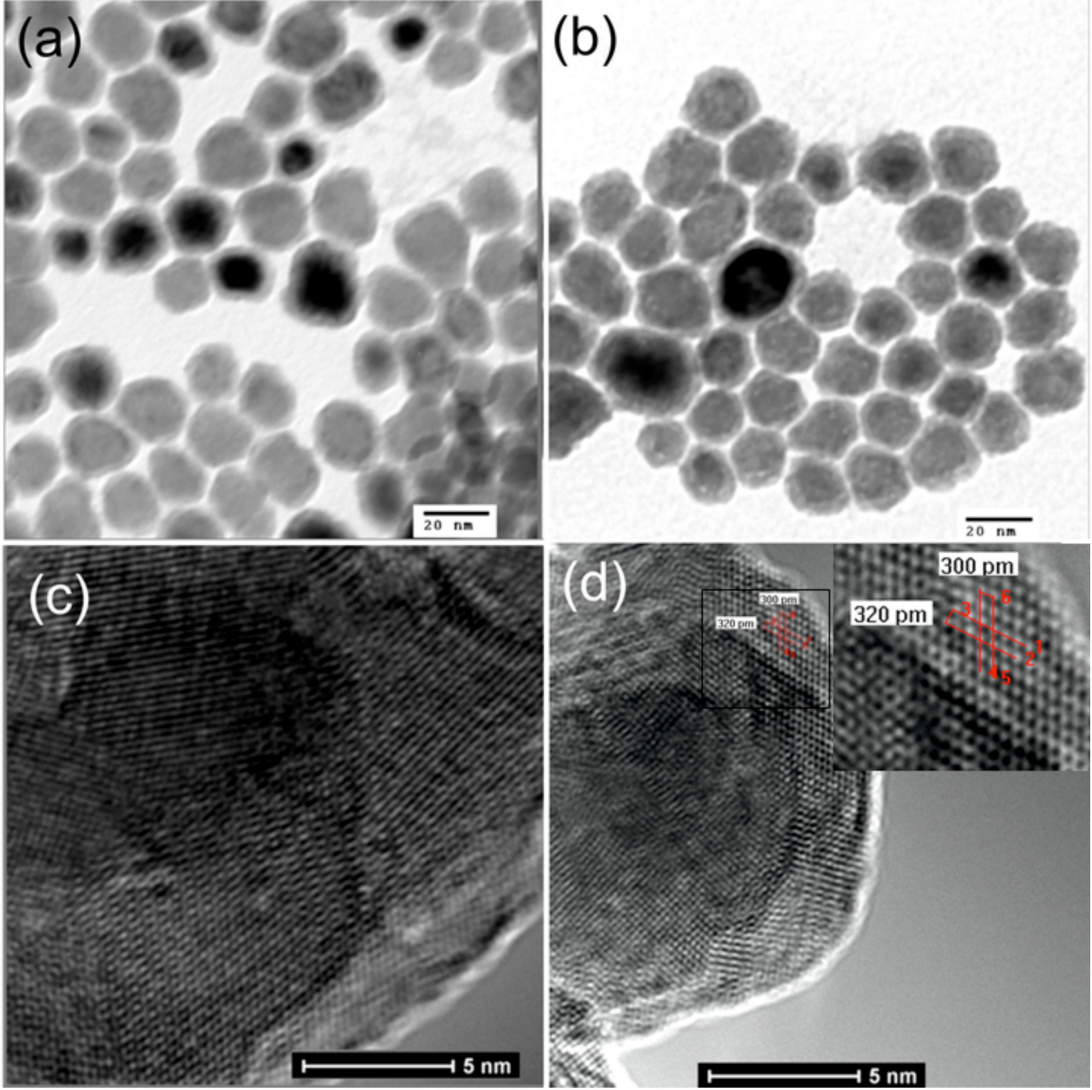
TEM of the core/shell Fe/Fe_3_O_4_ nanoparticles: (a) freshly synthesized MNPs; (b) MNP-SN38; (c) HRTEM of MNP; (d) HRTEM of MNP-SN38. (Note that the dark spots in a and b result from the presence of multiple layers.)

Figure 1a shows a low-resolution transmission electron microscope (TEM) image of the nanoparticles. The image reveals that the nanoparticles are roughly spherical, and a core/shell structure of the nanoparticles is clearly demonstrated. The average Fe(0) core diameter is 12 ± 0.5 nm and the thickness of the Fe_3_O_4_ shell is around 1.5 ± 0.5 nm. Exchange of the oleylamine/HDA ligands with the dopamine-based hydrophilic ligands **7** and **13** effectively renders the nanoparticle water-soluble. Figure 1b shows the TEM image of SN38-loaded Fe/Fe_3_O_4_ nanoparticles from PBS (pH 7.4) dispersion. Comparing the two TEM images, negligible changes on both, shape and size of the nanoparticles could be discerned. As revealed by high-resolution transmission electron microscopy (HRTEM) (Figure 1c and Figure 1d), the SN38 loaded Fe/Fe_3_O_4_ nanoparticles are crystalline with distinct lattice fringes.

**Figure 2:**
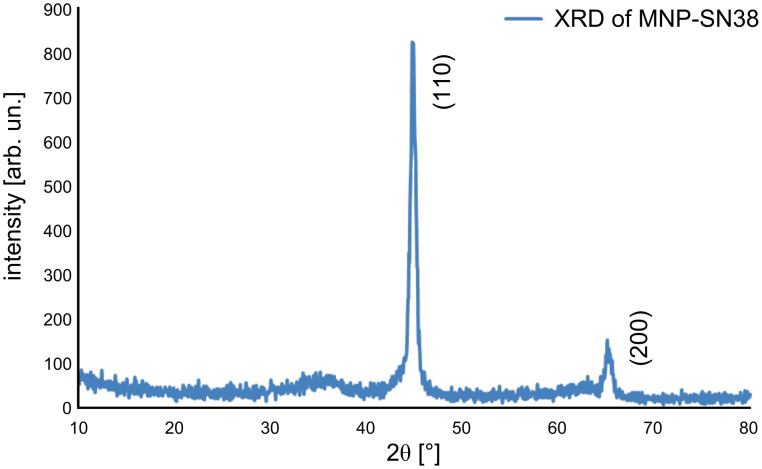
Powder XRD patterns of MNP-SN38.

Figure 2 shows the powder X-ray diffraction (XRD) pattern of SN38-loaded Fe/Fe_3_O_4_ magnetic nanoparticles. Highly crystalline structures were confirmed. The XRD peaks at 2θ = 44.7 and 65.1° correspond to (110) and (200) lattice-plane spacings of bcc-Fe [38,50]. No Fe_3_O_4_ diffraction peaks are observed due to their small crystal domains. It has been found that this nanoplatform is very robust against oxidation. The XRD patterns remained virtually unchanged even after the MNP-SN38 was exposed to air for two weeks at room temperature.

**Figure 3:**
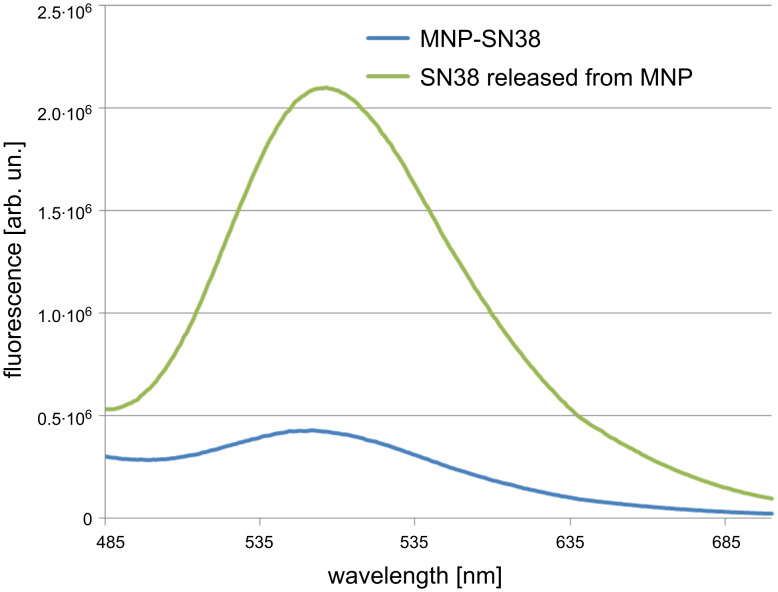
Fluorescence spectra of MNP-SN38 and free SN38 released from MNP.

The SN38-loaded Fe/Fe_3_O_4_ magnetic nanoparticles can be easily dispersed in pH 7.4 PBS buffer (up to 30 mg/mL). The dynamic light scattering (DLS) shows that the hydrodynamic diameter of the nanoparticles in water is about 95 nm, indicating that some level of aggregation occurred in the aqueous media, but overall the nanoparticles are monodisperse with a narrow size distribution (polydispersity <0.20) (Supporting Information File 1, Figure S1). Since the MNP-SN38 platform will be loaded on Mo/Ma cells for target delivery, the low-level aggregation of the MNP-SN38 will not cause such a problem as direct IV injection does. The zeta potential measurement carried out in deionized water at pH 7 demonstrates that the nanoparticles bear positive charges on the surface, with a value of 27.8 mV (Supporting Information File 1, Figure S2). This value is close to the threshold of 30 mV, which is considered as stable for nanoparticles [51]. Fluorescence spectra of MNP-SN38 and SN38 released from the same amount of MNP-SN38 were presented in Figure 3. Significant fluorescence quenching was observed for SN38 tethered on the surface of Fe/Fe_3_O_4_ magnetic nanoparticles, indicating the close proximity of SN38 to the magnetic nanoparticle. We also performed UV–vis characterization of the MNP-SN38 in PBS solution, but the absorption of SN38 was not observed due to overlapping with the broad MNP absorption.

### Loading content of SN38 on Fe/Fe_3_O_4_ nanoparticles

The release of SN38 from the nanoparticles was carried out under basic conditions (pH 12) at elevated temperature (95 °C) in aqueous solution. Nanoparticles were removed by centrifugation at 8000 rpm, and free SN38 was extracted with DCM/methanol 4/1 solution three times after adjustment of the pH value of the supernatant to 3.0. Upon removal of the solvent, the obtained SN38 was redissolved in 10 mL stock solution of DCM/methanol 4/1 containing 70 μg/mL of anthracene. HPLC analysis demonstrated that SN38 and anthracene were nicely separated, with a retention time for SN38 of 7.581 min, and a retention time for anthracene of 16.749 min (Supporting Information File 1, Figure S3). SN38 standard solutions in the concentration range from 2.13 to 51.10 μg/mL were prepared in the same stock solution. A HPLC calibration curve for SN38 concentration versus relative peak area was constructed (Supporting Information File 1, Figure S4). By fitting the relative peak area with this calibration curve, the loading content of SN38 on the Fe/Fe_3_O_4_ nanoparticles was calculated to be 26 ± 3 mg/g.

#### AMF heating of MNP-SN38

Alternating magnetic field (AMF) heating of the SN38-loaded Fe/Fe_3_O_4_ in a low strength and frequency magnetic field generated by an alternating current, demonstrated the superior heating ability of the nanoparticles. Within five minutes, a temperature increase of more than 30 °C was achieved when exposing a dispersion of 2.0 mg nanoparticles in 2.0 mL of water to the alternating magnetic field (Supporting Information File 1, Figure S5). The specific absorption rate (SAR) is calculated to be 522 ± 40 W/g. We propose that the excellent heating capacity of magnetic nanoparticles is due to the presence of the Fe(0) core in this core/shell nanostructure, because Fe(0) has the highest saturation magnetization per unit mass among all the metal elements (σ_s_ = 218 Am^2^·kg^−1^ at 293 K) [52]. The heating caused by ultrasmall magnetic nanoparticles in an alternating magnetic field is due to the relaxation loss. Relaxation loss may be either Neel or Brownian. In Neel relaxation, the nanoparticles do not move, but the direction of magnetization inside the particles rotates. In Brownian relaxation, the whole particle rotates against resistance due to the viscosity of the surrounding medium. For the localized magnetic hyperthermia application using our core/shell MNP-SN38 nanoplatform, we suggest that the heat generation is contributed by a combination of both Neel relaxation loss and Brownian relaxation loss [29]. The superior heating capacity of our nanoparticles permits both lower concentrations and shorter AMF exposure times during magnetic hyperthermia treatment.

#### Loading and toxicity of MNP-SN38 on cells

**Figure 4:**
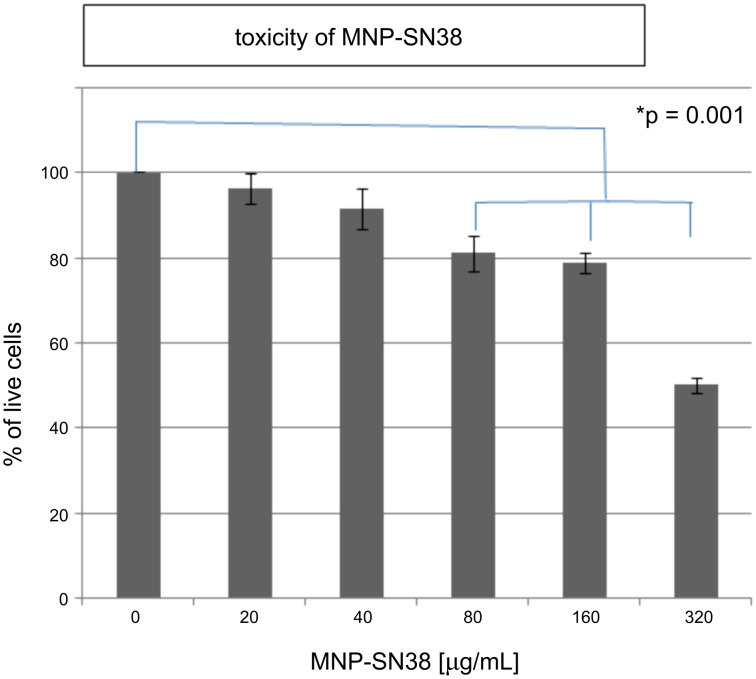
Toxicity of MNP-SN38 on double stable Mo/Ma after 24 h of loading; the MTT assay was performed for cell viability, and cell viability of 100% is considered in the case of the control group.

**Figure 5:**
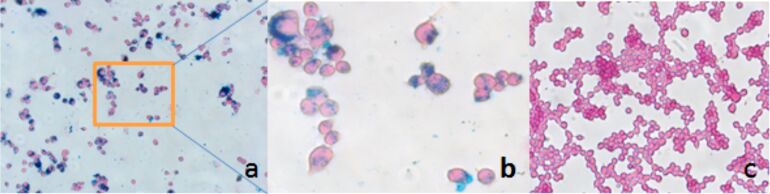
Double-stable Mo/Ma loaded with MNP-SN38 320 g/mL(medium). a: Prussian blue staining and counter stained by nuclear fast red 20×; b: 40×; c: control double-stable Mo/Ma Prussian blue stained and counter stained by nuclear fast red 20× (all images were taken in bright field).

We selected tumor-homing cells, double-stable Mo/Ma as a model cell to test the loading and toxicity of these nanoparticles. To determine the optimal loading of MNP-SN38, first the toxicity of MNP-SN38 for the double-stable Mo/Ma was determined. Different concentrations of nanoparticles were taken up by double-stable Mo/Ma cells over 24 h; the nanoparticle-concentration ranged from 0 to 320 μg/mL MNP-SN38 in fresh medium. After 24 h, the inhibition of cell proliferation was measured by using the MTT assay (Figure 4). We found only 20% of inhibition of cell proliferation at 160 μg/mL. Our aim is the loading of high payloads onto each delivery cell without causing a high level of necrosis or apoptosis of the delivery cells. Even a loading of 320 μg/mL of nanoparticles in the medium inhibits only 50% of the cell proliferation. We are also interested in the long term toxicity without activating the prodrug and changing cell morphology after loading. We have found that these nanoparticles showed no further toxicity even after five days (Figure 5). The successful loading of MNP-SN38 was confirmed by Prussian blue staining [53]. Nanoparticle-loaded cells feature blue dots, indicating the presence of iron. In contrast, no blue dots were observed in control cells. Moreover, after loading into the Mo/Ma cells, nanoparticles remained separated even after five days. This demonstrated the robust stability of the MNP-SN38 platform under physiological conditions, which is highly desired for a cell-based delivery system. We have established that when Mo/Ma cells are used as carriers, the tumor-homing process takes about one day. Their robust stability will ensure the integrity of the MNPs after delivery to the tumor site.

**Figure 6:**
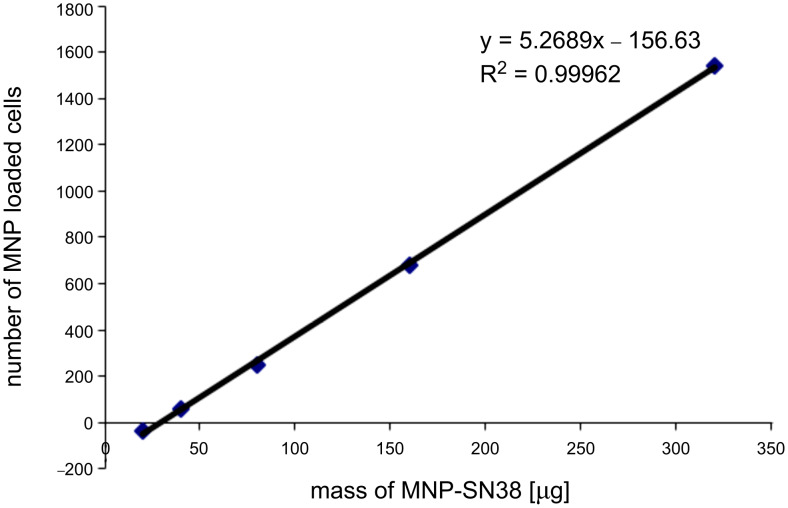
Flow cytometry of MNP-SN38 loaded double-stable Mo/Ma after 24 h. Side scatter was used to measure loading of nanoparticles in cells. Concentrations of 0–320 µg/mL of MNP-SN38 were loaded and allowed 24 h for loading.

**Figure 7:**
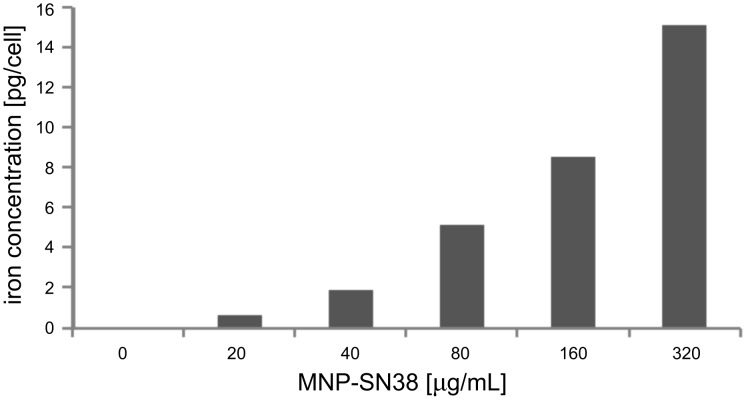
Iron concentration per double-stable Mo/Ma cell loaded with different concentrations of MNP-SN38.

The uptake efficiency of MNP-SN38 platform by the double-stable Mo/Ma was determined by flow cytometry. Different concentrations of nanoparticles were loaded into the cells over 24 h, by using nanoparticle concentrations between 0 and 320 μg/mL in culture medium. After 24 h of loading, the cells were washed three times with 1× PBS, lifted and analyzed by flow cytometry. The “side scatter” function was used to determine the loading of nanoparticles in the cells and compared to the “side scatter” of control cells (Figure 6). The uptake of nanoparticles by Mo/Ma cells correlates with the amount of nanoparticles loaded in their culturing medium, indicating that the MNP-SN38 platform can be easily loaded in the delivery cells in defined concentrations. The iron content of the nanoparticles, as well as the concentration of iron in nanoparticle-loaded cells was determined by using the ferrozine assay. A mass of 1.0 mg of nanoparticles contained 0.427 mg of iron, indicating that this amount of iron would be high enough for alternating magnetic field hyperthermia in combination with chemotherapy [54]. The MTT assay indicated that 8 pg of iron can be easily loaded in each cell (20% inhibition of cell proliferation) (Figure 4 and Figure 7). It is even possible to load 16 pg of iron in each cell (with 50% inhibition of cell proliferation) indicating that 2.1·10^−15^ mol of SN38 can be easily loaded in each delivery cell.

To test the release of SN38 by the self-contained Tet-On Advanced system, Mo/Ma cells were plated in a 24-well plate at a density of 300,000 cm^−2^, and incubated overnight at 37 °C to become 70% confluent. The next day, the medium was removed, and wells of the plate were divided into three groups evenly, each group containing eight replications. SN38-loaded Fe/Fe_3_O_4_ nanoparticles in fresh medium at 80, 160, and 320 µg/mL were added to group 1, group 2 and group 3, respectively. After incubation for 24 h, the medium was removed and the cells were washed with fresh Mo/Ma medium. Then 1 μg/mL doxycycline containing medium was added to half of the replications in each group, and to the other half only fresh medium was added. After incubation for three days, the medium in each group with and without doxycycline was collected and centrifuged for 5 min at 1000 rpm to remove cell debris. The aqueous phases were extracted with methylene chloride three times, and the combined methylene chloride phases were dried over anhydrous Na_2_SO_4_. After concentration of the volume to 500 μL, each sample was subjected to HPLC analysis. No SN38 was observed by HPLC in the control groups; in contrast, a significant peak corresponding to the SN38 retention time was visible in HPLC for groups with added doxycycline (Supporting Information File 1, Figure S6), indicating the successful release of SN38 by the Tet-On Advanced system.

## Conclusion

**Scheme 3:**
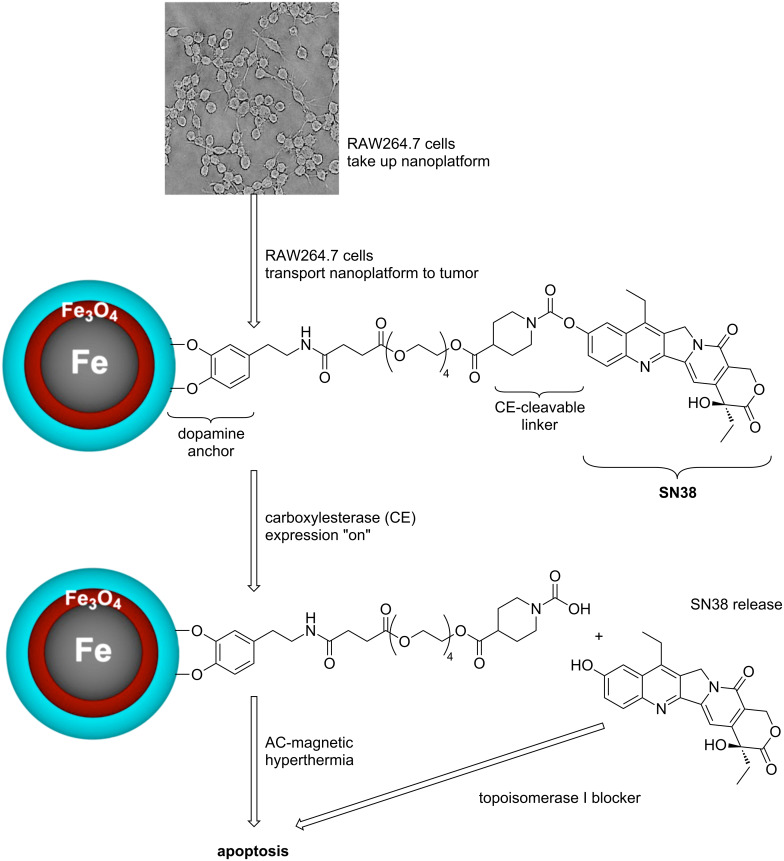
RAW264.7 cell (monocyte/macrophage) delivered thermochemotherapy.

We have developed a nanoplatform that will potentially permit the treatment of cancer by a combination of magnetic hyperthermia and chemotherapy (thermochemotherapy) after targeted delivery by double-stable RAW264.7 monocyte/macrophage-like cells (Mo/Ma). A carboxylesterase-cleavable irinotecan-like SN38 prodrug was synthesized and attached to Fe/Fe_3_O_4_ magnetic nanoparticles. The prodrug concentration that was chemisorbed via dopamine-anchors to the Fe_3_O_4_ outer layer of the core/shell nanoparticles was 26 ± 3 mg/g. The MNP-SN38 nanoplatform showed efficient heating ability in an alternating magnetic field (SAR = 522 ± 40 W/g). In accordance with the design of the dopamine-anchored SN38 prodrug, the nanoplatform demonstrated minimal cytoxicity for tumor-homing Mo/Ma cells. These cells feature a Tet-On Advanced system for intracellular carboxylesterase (InCE) expression. Upon addition of doxycycline, SN38 was released from the nanoplatform, as evidenced by HPLC analysis. Therefore, this nanoplatform can be potentially used as a multipurpose agent in cancer therapy through highly localized magnetic hyperthermia and triggered release/activation of the chemotherapeutic drug SN38 at the cancer site. Using the synergy between targeted chemotherapy and hyperthermia will make cell-delivered anticancer treatment a viable option. Scheme 3 summarizes this approach to Mo/Ma-cell-delivered thermochemotherapy.

## Supporting Information

Detailed experimental procedures, spectroscopic characterizations, DLS and zeta-potential measurements, as well as HPLC analysis are provided.

File 1 Detailed experimental data.
