# Supplementary material for: Magnetic-Fe/Fe3O4-nanoparticle-bound SN38 as carboxylesterase-cleavable prodrug for the delivery to tumors within monocytes/macrophages
Source: Beilstein J Nanotechnol. 2012 Jun 13;3:444–55. doi: 10.3762/bjnano.3.51 (PMC3388369; doi:10.3762/bjnano.3.51)
Supplement: File 1 — Detailed experimental data. [file Beilstein_J_Nanotechnol-03-444-s001.pdf]

Supporting Information

for

Magnetic Fe/Fe_3O_4 nanoparticle-bound SN38 as carboxylesterase-cleavable prodrug for the delivery to tumors within monocytes/macrophages

Hongwang Wang[1*], Tej B. Shrestha[2], Matthew T. Basel[2], Raj K. Dani[1], Gwi-Moon Seo[2], Sivasai Balivada[2], Marla M. Pyle[2], Heidy Prock[1], Olga B. Koper[3], Prem S. Thapa[4], David Moore[4], Ping Li[1], Viktor Chikan[1], Deryl L. Troyer[2*] and Stefan H. Bossmann[1*]

Address: [1]Kansas State University, Department of Chemistry, CBC 201, Manhattan, KS 66506; [2]Kansas State University, Anatomy&Physiology, Coles 228, Manhattan, KS 66506; [3]Battelle Memorial Institute, 505 King Ave., Columbus, OH 43201 and [4]University of Kansas, KU Microscopy & Analytical Imaging Laboratory, 1043 Haworth, Lawrence, KS 66045

Email: Hongwang Wang* - hongwang@ksu.edu; Deryl L. Troyer* - troyer@vet.k-state.edu; Stefan H. Bossmann* - sbossman@ksu.edu

*Corresponding author

Detailed experimental data

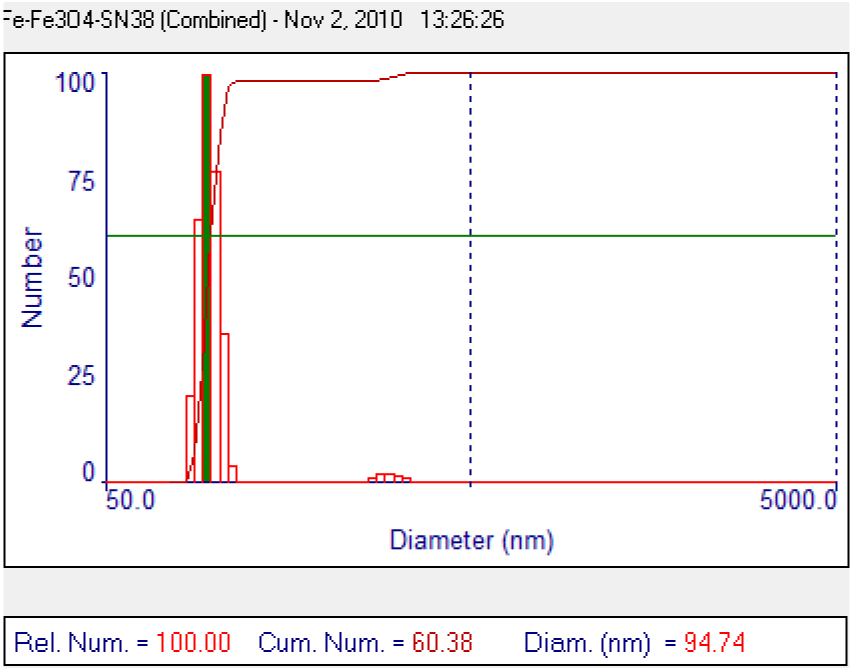

Figure S1: DLS measurement of the MNP-SN38.

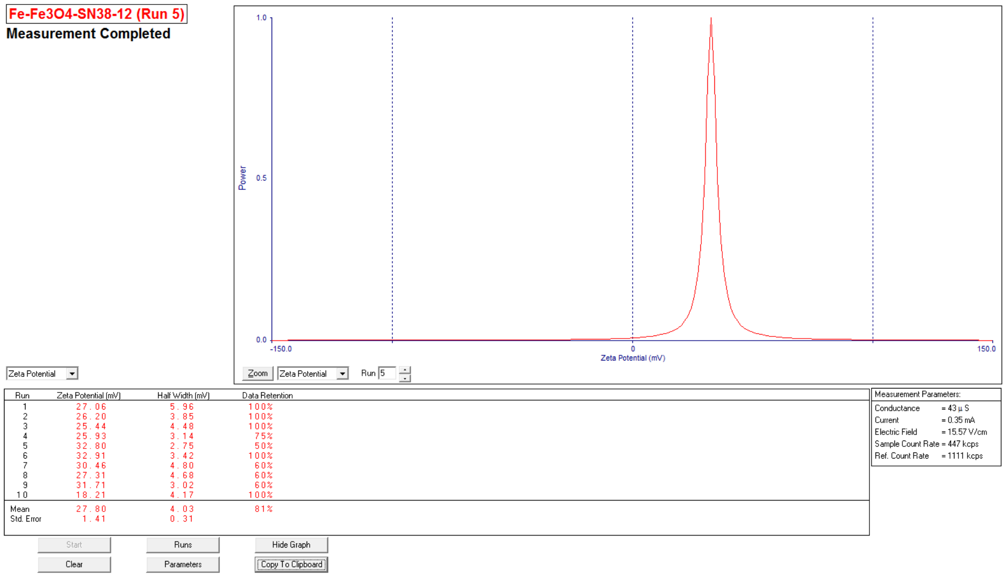

Figure S2: Zeta potential measurement of MNP-SN38.

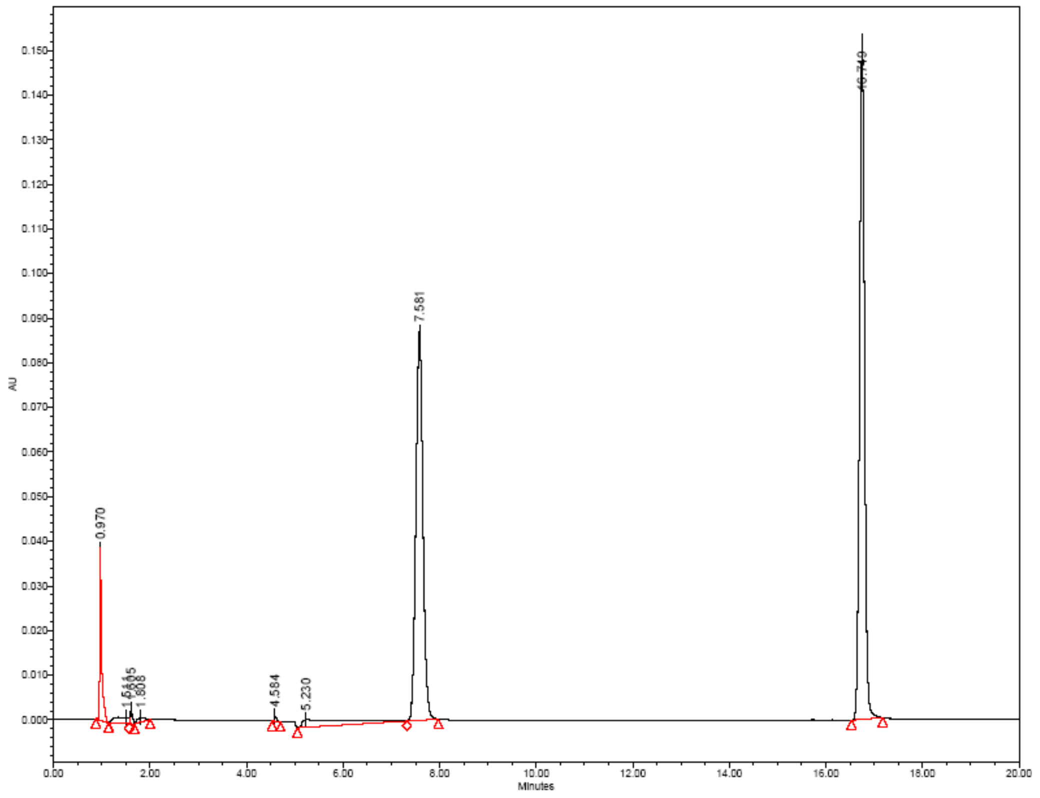

Figure S3: HPLC analysis of SN38 released from MNPs (peak at 7.581 min is SN38, peak at 16.749 min is anthrancene which was used as internal standard).

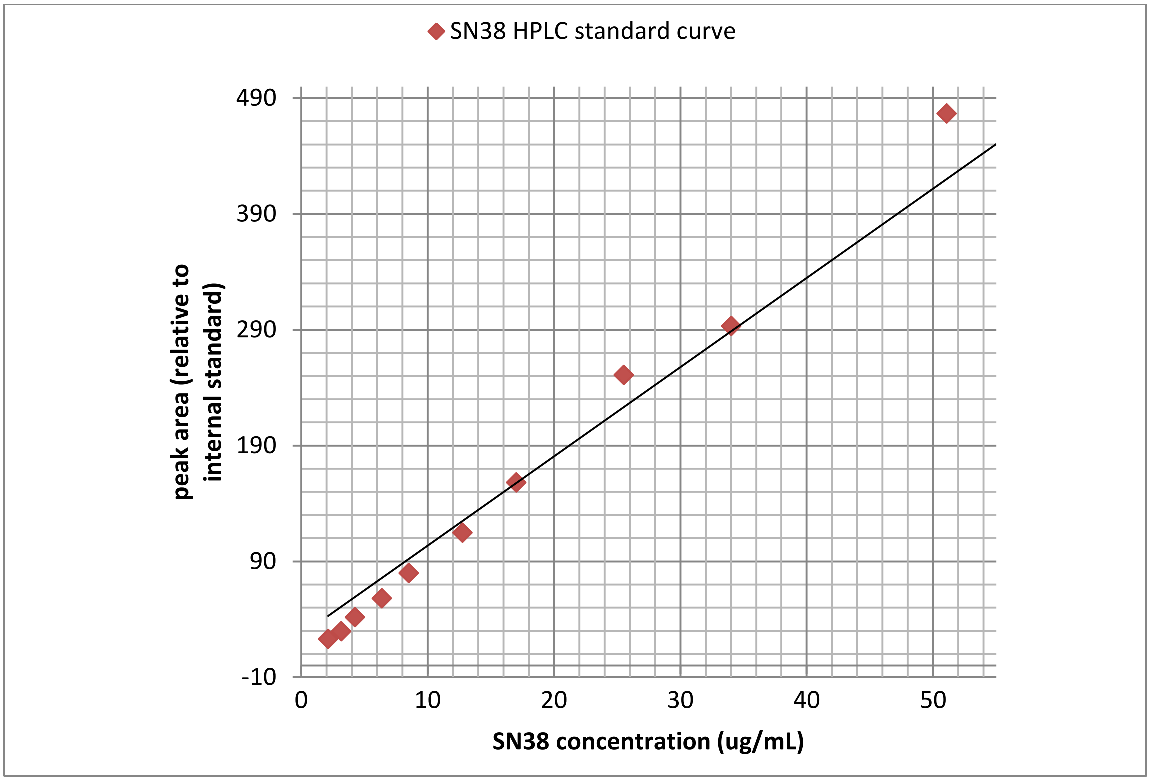

Figure S4: SN38 HPLC standard curve. (SN38 concentration range from 2.13 microgram/mL to 51.10 microgram/mL. Anthrancene (70 microgram/mL) as internal standard. Anthrancene's peak area was normalized to 100. SN38's peak area was calculated relative to normalized internal standard.)

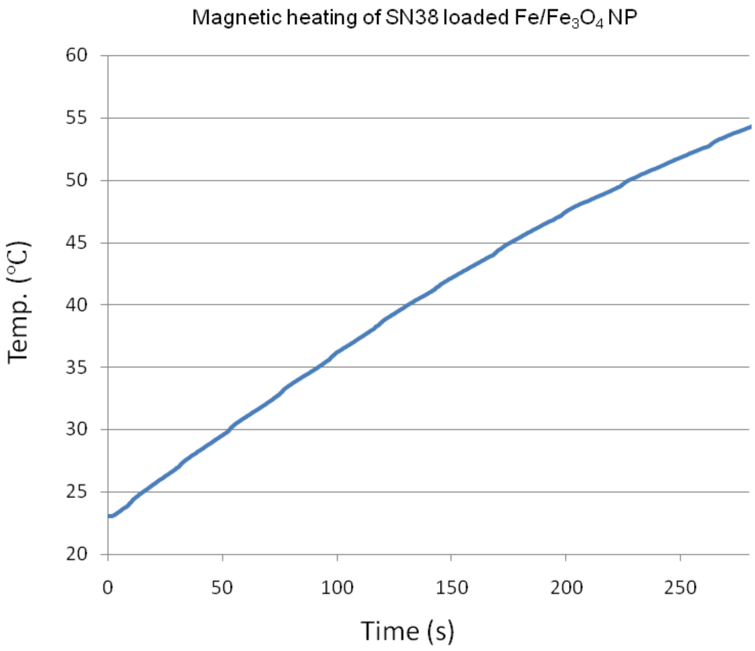

Figure S5: AC-magnetic heating curve of the MNP-SN38.

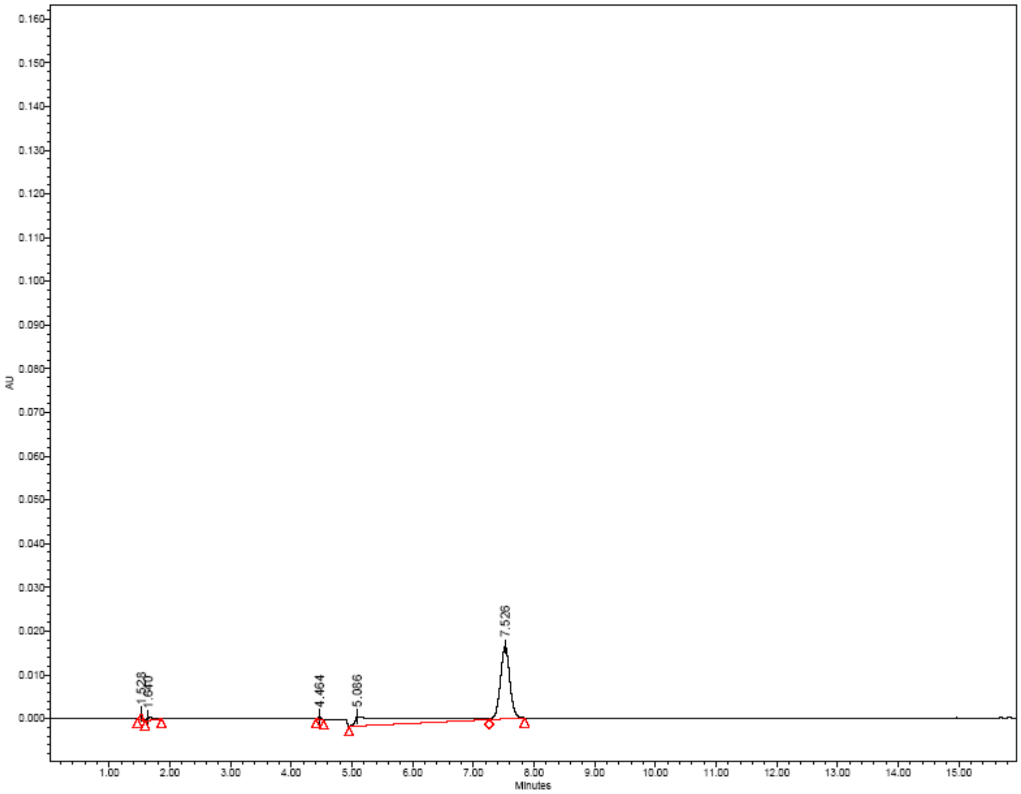

Figure S6: HPLC analysis of SN38 released from Mo/Ma by adding doxycycline.

Experimental

1. Synthesis of core/shell Fe/Fe_3O_4 nanoparticles

Iron nanoparticles were prepared by extensive modification of a literature procedure described by Lacroix et al. [1]. 20 mL octadecene, 0.3 mL oleylamine and 0.277 g hexadecylammonium chloride (HAD·HCl) were added to a 100 mL Schlenk flask, and the mixture was degassed at 120 °C for 30 min under vigorous stirring. After refilling the Schlenk flask with argon, the temperature was raised to 180 °C. 0.7 mL $Fe(CO)_5$ was added under argon. The solution turned black within 3 min. and was kept at 180 °C for further 30 min before allowed to cool to room temperature. Under argon protection, the

supernatant was decanted. Iron nanoparticles accumulated on the stir bar were washed with hexane and ethanol. The product was dried in vacuum and stored at RT for further use. Based on iron, the yield of the reaction is 88%.

2. Linking SN38 to dopamine-based Fe/Fe_3O_4 nanoparticle anchor

Boc-protection of Dopamine [2]

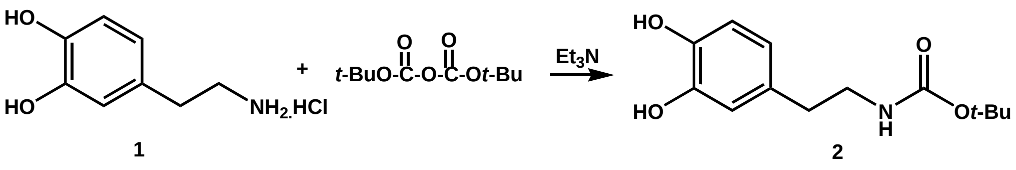

A solution of dopamine **1** (310 mg, 1.63 mmol) in methanol (8 mL) was stirred under N_2 for 5 minutes. TEA (1.8 mmol) was added followed by Boc-anhydride (393 mg, 1.8 mmol). The mixture was stirred under N_2 for 12 hours and the solvent was removed under reduced pressure. The remaining residue was dissolved in 40 mL CH_2Cl_2 and washed with 1 N HCl (3×5 mL) and brine (5 mL). The organic layer was dried over anhydrous Na_2SO_4. After filtration, the organic phase was kept at −5 °C for 3 hours. A white precipitate came out as product **2** and collected by filtration. (98% yield). ^{1}H NMR (DMSO-d_6) δ: 1.73 (s, 9H); 2.48 (t, 2H); 3.02 (q, 2H); 6.40 (d, 1H); 6.54 (s, 1H); 6.61 (d, 1H); 6.83 (t, 1H); 6.85 (s, 1H); 6.76 (s, 1H).

Benzyl-protection of Boc-dopamine [3]

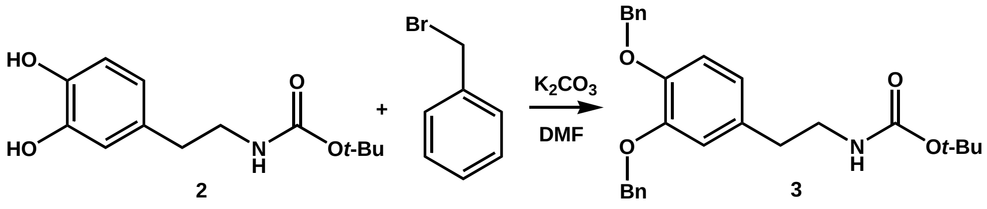

3.47 g Boc-protected dopamine **2** was dissolved in 100 mL DMF. 12.6 g K_2CO_3 was added and the system was protected under N_2. 4.69 g (2 eq.) benzyl bromide was added drop wise. The mixture was stirred at room temperature for 24 hours without light. The solid was removed by filtering through a short pad of celite and the filter-cake was washed with ether (3×100 mL). The combined filtrate and washing solution were washed with ice-water (3×50 mL) and brine (15 mL). The organic layer was dried over anhydrous Na_2SO_4 and concentrated to 150 mL. After setting at −5 °C for 5 hours, white precipitate came out as product **3** and was collected by vacuum filtration. (93% yield). ^{1}H NMR ($CDCl_3$) δ: 1.45 (s, 9H); 2.70 (t, 2H); 3.31 (q, 2H); 4.49 (s, 1H); 5.15 (d, 4H); 6.71 (d, 1H); 6.80 (s, 1H); 6.88 (d, 1H); 7.32 (t, 2H); 7.37 (t, 4H); 7.45 (d, 4H).

Deprotection of the Boc-group [3]

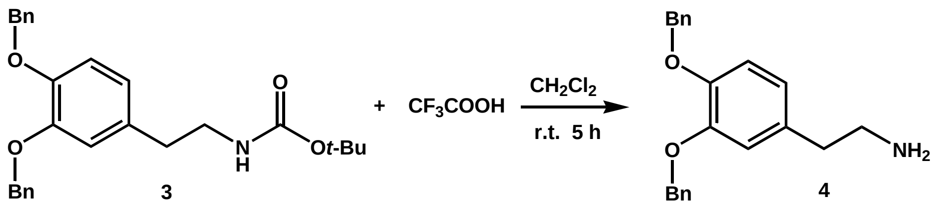

4.3g Bn-Boc-dopamine **3** was dissolved in 150 mL 5% TFA CH_2Cl_2 solution and stirred at room temperature for 5 hours. The solvent was removed in vacuum and clear oil was

obtained as product **4**. (100% yield). ^{1}H NMR ($CDCl_3$) δ: 2.79 (t, 2H); 3.08 (m, 2H); 5.11 (s, 4H); 6.68 (d, 1H); 6.75 (s, 1H); 6.90 (d, 1H); 7.32 (t, 2H); 7.35 (t, 4H); 7.42 (d, 4H). ^{13}C NMR ($CDCl_3$) δ: 32.90; 41.85; 71.50; 72.00; 115.60; 116.25; 122.30; 127.60; 127.85; 128.35; 128.45; 128.63; 128.85; 136.70; 136.85; 148.45; 149.00; 160.88; 161.20; 161.58; 161.90.

Amide Formation [3]

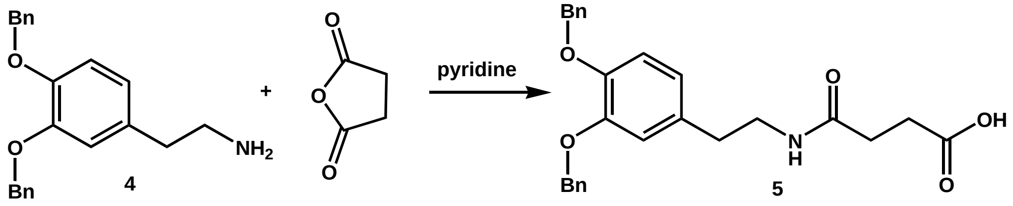

1.43 g Bn-dopamine **4** and 0.43 g succinic anhydride (1/1 ratio) were dissolved in 6 mL pyridine. The solution was stirred at room temperature for 5 hours. The solvent was removed by co-evaporation with toluene (toluene 5×5 ml). White solid was obtained and washed with CH_2Cl_2 for 3 times. After drying in vacuum, 1.4 g product **5** was obtained. (89% yield). ^{1}H NMR (DMSO-d_6) δ: 2.29 (t, 2H); 2.42 (t, 2H); 2.60 (t, 2H); 3.21 (q, 2H); 5.09 (d, 4H); 6.71 (d, 1H); 6.94 (s, 1H); 6.96 (d, 1H); 7.32 (t, 2H); 7.38 (d, 4H); 7.45 (t, 4H); 7.90 (t, 1H); 12.08 (s, 1H). MS-ESI+: m/z 434.2. Molecular weight calculated for 433.5**.**

Bn-Dop-Tetraethylene glycol

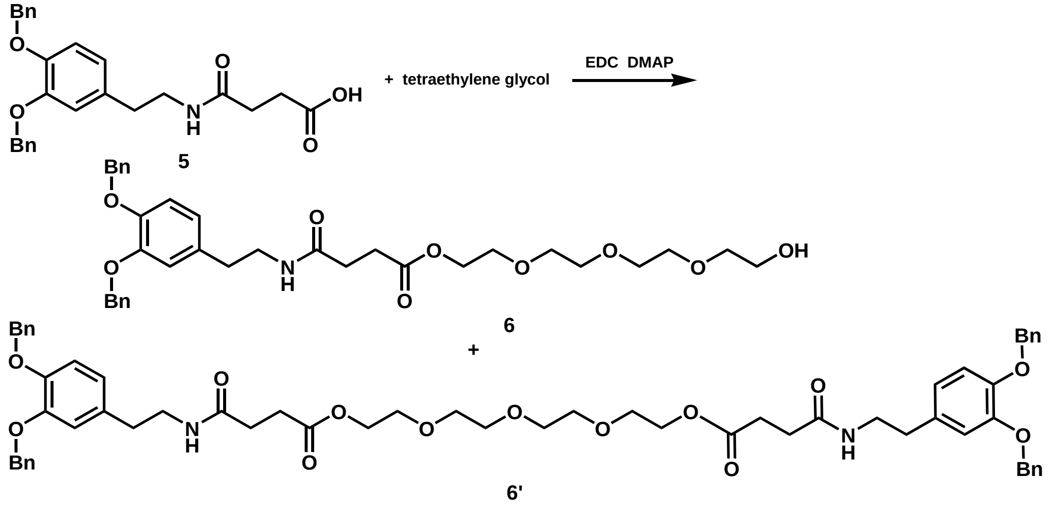

0.964 g dopamine-based carboxylic acid **5** and 0.426g EDC (1/1 ratio) were dissolved in 100 mL CH_2Cl_2 and stirred at room temperature for 10 minutes. 0.433 g tetraethylene glycol was added followed by 5 mg DMAP. After stirring for 12 hours at room temperature, the organic phase was washed with 10% H_3PO_4 solution (3×10 mL), water (3×10 mL) and brine (10 mL). The organic phase was dried over anhydrous $MgSO_4$. After removing the solvent in vacuum, the residue was loaded on column and eluted with 1/1 acetone/methylene chloride. 0.77 g ideal product **6** was obtained. (79% yield). 0.21 g side product **6’** was isolated. ^{1}H NMR for **6** ($CDCl_3$) δ: 2.39 (t, 2H); 2.57 (t, 1H); 2.70 (q, 4H); 3.44 (q, 2H); 3.60 (t, 2H); 3.65 (broad 12H); 4.24 (t, 2H); 5.15 (d, 4H); 5.74 (t, 1H); 6.71 (d, 1H); 6.81 (s, 1H); 6.89 (d, 1H); 7.31 (t, 2H); 7.37 (t, 4H); 7.46 (d, 4H). MS-ESI+: m/z 610.4. Molecular weight calculated for 609.3. ^{1}H NMR for **6’** ($CDCl_3$) δ: 2.37 (t, 4H); 2.67 (m, 8H); 3.42 (q, 4H); 3.63 (s, 8H); 3.67 (t, 4H); 4.22 (t, 4H); 5.15 (d, 8H); 5.70 (t, 2H); 6.70 (d, 2H); 6.80 (s, 2H); 6.88 (d, 2H); 7.31 (t, 4H); 7.36 (t, 8H); 7.45 (d, 8H). MS-ESI+: m/z 610.4. Molecular weight calculated for 609.3.

Preparation of Ligand I

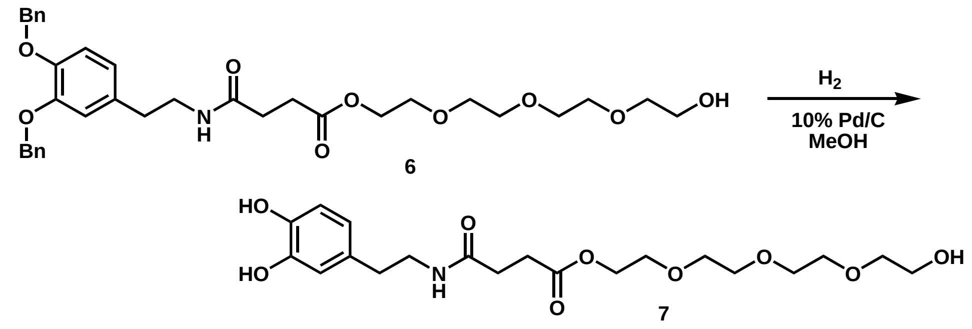

0.34 g Bn-dopamine-based tetraethylene glycol **6** was dissolved in 50 mL methanol. 77 mg Pd/C was added under N_2. After evacuating three times, 1 atm. H_2 was applied and the mixture was stirred for 24 hours at room temperature. The catalyst was removed by filtering through a short pad of celite. After removing solvent in vacuum, 0.23 g product **7** was obtained. (100% yield). ^{1}H NMR (DMSO-d_6) δ: 2.33 (t, 2H); 2.48 (q, 2H); 3.15 (broad multiplet, 4H); 3.41 (t, 2H); 3.49 (t, 2H); 3.51 (broad multiplet, 8H); 3.59 (t, 2H); 4.11 (t, 2H); 6.41 (d, 1H); 6.55 (s, 1H); 6.61 (d, 1H). MS-ESI$^+$: *m/z* 430.4. Molecular weight calculated for 429.4.

Bn-dop-tetraethylene-glycol-piperidine-Fmoc

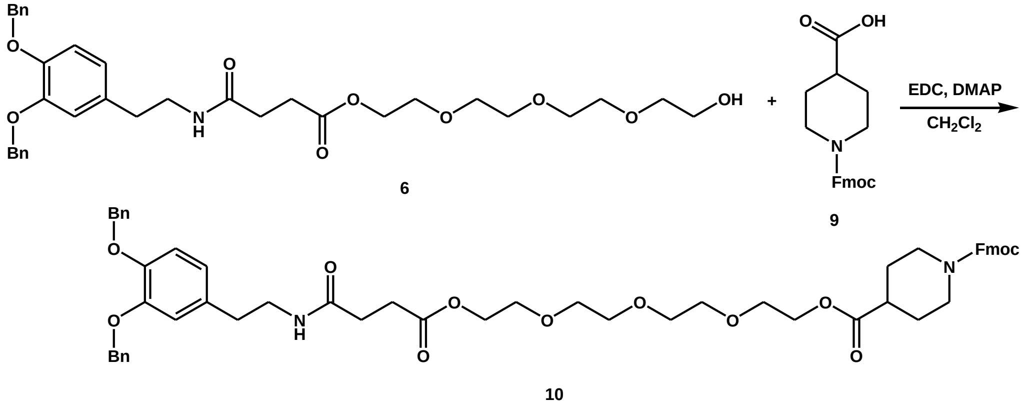

0.775 g of **6** and 0.452 g of **9** were dissolved in 30 mL methylene chloride, stirred at room temperature for 10 minutes. 0.292 g of EDC.HCl and 0.155 g of DMAP were added to the solution. After stirring at room temperature for 12 hours, 20 mL of methylene chloride was added. The reaction mixture was washed with 10% (v/v) H_3PO_4 water solution (3 ×10 ml), water (3 ×10 ml) and brine (10 ml). The organic phase was dried over anhydrous $MgSO_4$ for 2 hours. Solvent was removed in vacuum. Product **10** was purified by column chromatography (Silica gel, eluted with 6/1 methylene chloride/acetone solvent mixture). (86% yield). ^{1}H NMR for 8 ($CDCl_3$) δ: 1.65 (broad singlet, 2H); 1.90 (broad singlet, 2H); 2.38 (t, 2H); 2.53 (m, 1H); 2.66-2.71 (m, 4H); 2.92 (broad singlet, 2H); 3.41-3.46 (dd, 2H); 3.65 (d, 10H); 3.67-3.71 (quintet, 4H); 3.99-4.08 (m, 2H); 4.22-4.27 (m, 5H); 4.41 (m, 2H); 5.14 (s, 2H); 5.16 (s,2H); 5.68 (t, 1H); 6.69 (dd, 1H); 6.80 (d, 1H); 6.88 (d, 1H); 7.29-7.46 (m, 14H); 7.58 (d, 2H); 7.77 (d, 2H). ^{13}C NMR ($CDCl_3$) δ: 27.60; 27.74; 29.43; 30.89; 35.02; 40.42; 40.67; 43.10; 47.24; 63.59; 63.69; 63.87; 67.25; 68.93; 69.02; 70.44; 70.53; 70.81; 71.10; 71.29; 115.19; 115.50; 119.93; 121.46; 124.89; 126.98; 127.22; 127.26; 127.62; 127.71; 128.40; 132.13; 137.18; 137.28; 141.23; 143.91; 147.48; 148.84; 155.06; 171.41; 172.84; 174.27; 178.30. MS-ESI+: m+Na/z 965.3. Molecular weight calculated for 942.6.

Deprotection of Fmoc

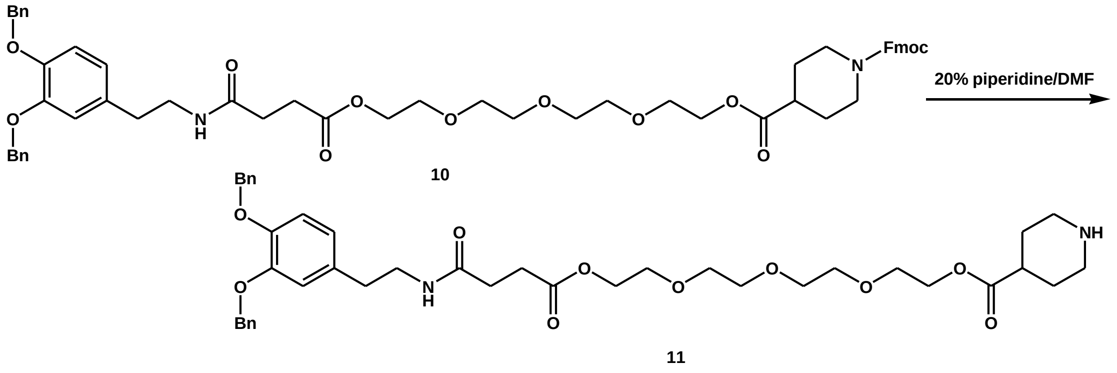

0.452 g of **10** was dissolved in 6 mL of 20% piperidine/DMF solution, after stirring at room temperature for 30 minutes; solvent was removed under high vacuum (oil pump) at 40 °C. The solid residue was washed with hexane to remove the side product. 0.258 g clear oil was obtained as pure product after drying under vacuum. (78% yield) ^{1}H NMR for **11** ($CDCl_3$) δ: 1.60-1.65 (m, 2H); 1.88-1.92 (m, 2H); 2.38 (t, 2H); 2.60-2.72 (m, 5H); 3.07-3.12 (m, 2H); 3.41-3.46 (dd, 2H); 3.65 (d, 10H); 3.67-3.71 (m, 4H); 4.22-4.25 (m, 4H); 5.15 (s, 2H); 5.17 (s,2H); 5.73 (t, 1H); 6.69 (dd, 1H); 6.80 (d, 1H); 6.88 (d, 1H); 7.31 (t, 2H); 7.37 (t, 4H); 7.46 (d, 4H). ^{13}C NMR ($CDCl_3$) δ: 24.80; 29.62; 31.02; 35.20; 38.04; 40.91; 42.92; 63.82; 63.96; 69.02; 69.11; 70.61; 70.62; 70.65; 70.73; 71.37; 71.57; 115.54; 115.87; 121.72; 127.44; 127.48; 127.88; 127.91; 128.57; 132.53; 137.41; 137.51; 147.71; 149.10; 171.59; 172.88; 173.07. MS-ESI+: m/z 721.1. Molecular weight calculated for 720.

Bn-dop-tetraethylene-glycol-piperidine-SN38 [4]

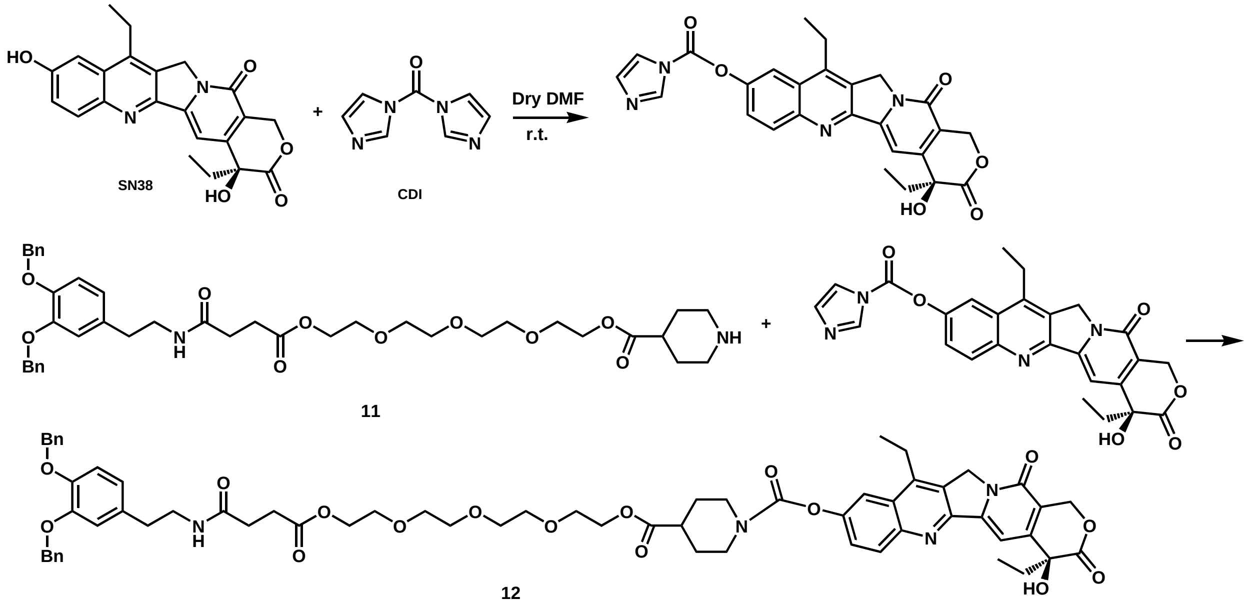

0.098 g of SN38 and 0.049 g of CDI were dissolved in 5 mL dry DMF, the reaction mixture was blanketed with dry argon and stirred at room temperature. The reaction progress was monitored by TLC. After 3 hours, most of the SN38 was converted to the activated intermediate. 0.182 g of **11** was added to the reaction mixture under the protection of dry argon. The reaction mixture was further stirred at room temperature for 48 hours. Solvent was removed under high vacuum. The product **12** was purified by column chromatography (Silica gel, eluted with 20/1 methylene chloride/acetone solvent mixture). The product was obtained as glassy foam. (77% yield). ^{1}H NMR for **12** ($CDCl_3$) δ: 1.04 (t, 3H); 1.40 (t, 3H); 1.78-1.96 (m, 4H); 2.02-2.09 (m, 2H); 2.38 (t, 2H); 2.60-2.72 (m, 6H); 3.04-3.28 (m, 4H); 3.44 (q, 2H); 3.62-3.67(m, 8H); 3.69 (t, 2H); 3.73 (t, 2H); 3.77 (s, 1H); 4.23 (t, 2H); 4.29 (t, 2H); 5.14 (d, 4H); 5.26 (s, 2H); 5.32 (d, 1H); 5.66 (t, 1H); 5.74 (d, 1H); 6.75 (dd, 1H); 6.80 (s, 1H); 6.83 (d, 1H); 7.30 (t, 2H); 7.36 (t, 4H);

7.42 (d, 4H); 7.58 (dd, 1H); 7.64 (d, 1H); 7.84 (d, 1H); 8.20 (d, 1H). ^{13}C NMR ($CDCl_3$) δ: 8.04; 14.22; 23.37; 29.70; 31.20; 31.81; 35.32; 40.84; 40.93; 49.61; 63.96; 63.98; 66.59; 69.25; 69.32; 70.62; 70.84; 70.86; 71.46; 71.65; 72.97; 98.09; 114.71; 115.60; 115.94; 118.60; 121.77; 127.50; 127.53; 127.98; 128.00; 128.67; 132.09; 132.46; 137.50; 137.59; 145.35; 147.25; 147.51; 147.88; 149.22; 150.37; 150.56; 151.87; 153.40; 157.87; 171.42; 173.09; 174.18; 174.36. MS-ESI+: m+K/z 1177.5. Molecular weight calculated for 1138.

Dop-tetraethylene-glycol-piperidine-SN38

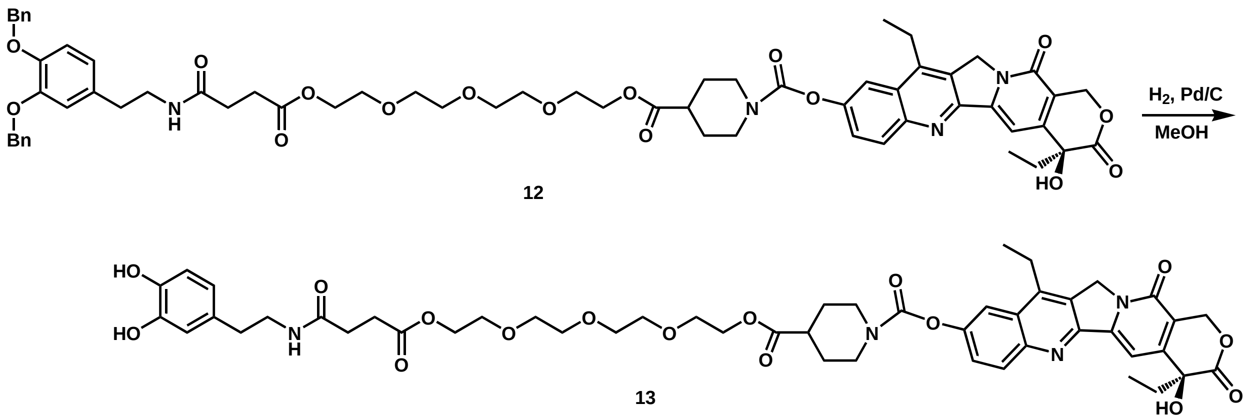

0.097 g of **12** was dissolved in 10 mL of THF, and 0.015 g of 10% Pd on activated carbon was added. The reaction mixture was stirred under 1 atm. hydrogen atmosphere at room temperature for 2 hours. The catalyst was removed by filtering through a short pad of celite. The filtrate was concentrated to dryness in high vacuum, a white solid was obtained as product **13**. (98% yield) ^{1}H NMR for **13** ($CDCl_3$) δ: 1.04 (t, 3H); 1.40 (t, 3H); 1.78-1.96 (m, 4H); 2.02-2.09 (m, 2H); 2.42 (t, 2H); 2.58-2.72 (m, 6H); 3.04-3.28 (m, 4H); 3.42 (q, 2H); 3.60-3.70(m, 10H); 3.73 (t, 2H); 3.92 (broad triplet, 1H); 4.18 (t, 2H); 4.29 (t, 2H); 5.24 (s, 2H); 5.32 (d, 1H); 5.74 (d, 1H); 6.02 (t, 1H); 6.52 (dd, 1H); 6.66 (s, 1H);

6.76 (d, 1H); 7.58 (dd, 1H); 7.66 (s, 1H); 7.84 (d, 1H); 8.22 (d, 1H). ^{13}C NMR ($CDCl_3$) δ: 8.04; 14.22; 23.37; 29.96; 30.54; 31.29; 31.82; 34.45; 34.71; 40.83; 40.99; 49.67; 63.93; 64.03; 66.53; 69.20; 69.34; 70.69; 70.79; 70.84; 73.02; 98.38; 114.79; 115.67; 116.10; 118.69; 120.83; 125.74; 126.05; 127.42; 127.73; 129.95; 131.23; 131.99; 136.00; 143.15; 144.34; 145.54; 147.13; 147.38; 150.55; 151.79; 153.50; 157.91; 171.99; 173.28; 174.08; 174.42. MS-ESI+: m+Na/z 981.3. Molecular weight calculated for 958.3.

NMR data

All spectra were acquired at explained in the general methods. Expansions are provided for enhanced clarity

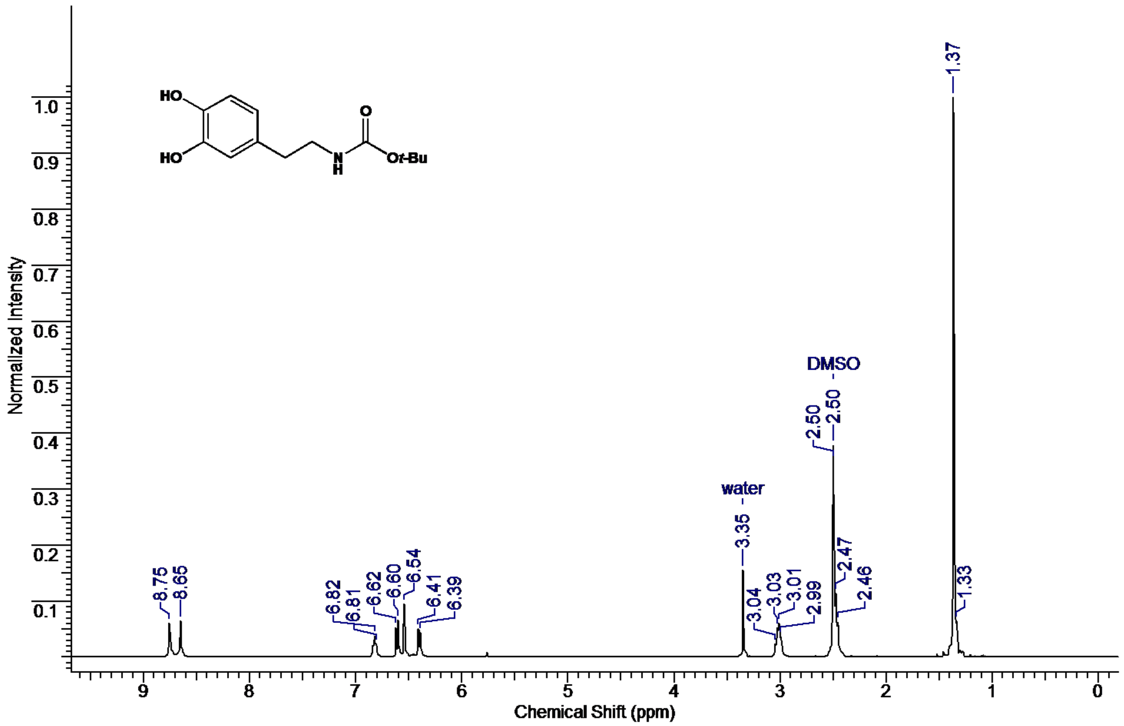

^{1}H NMR of compound 2

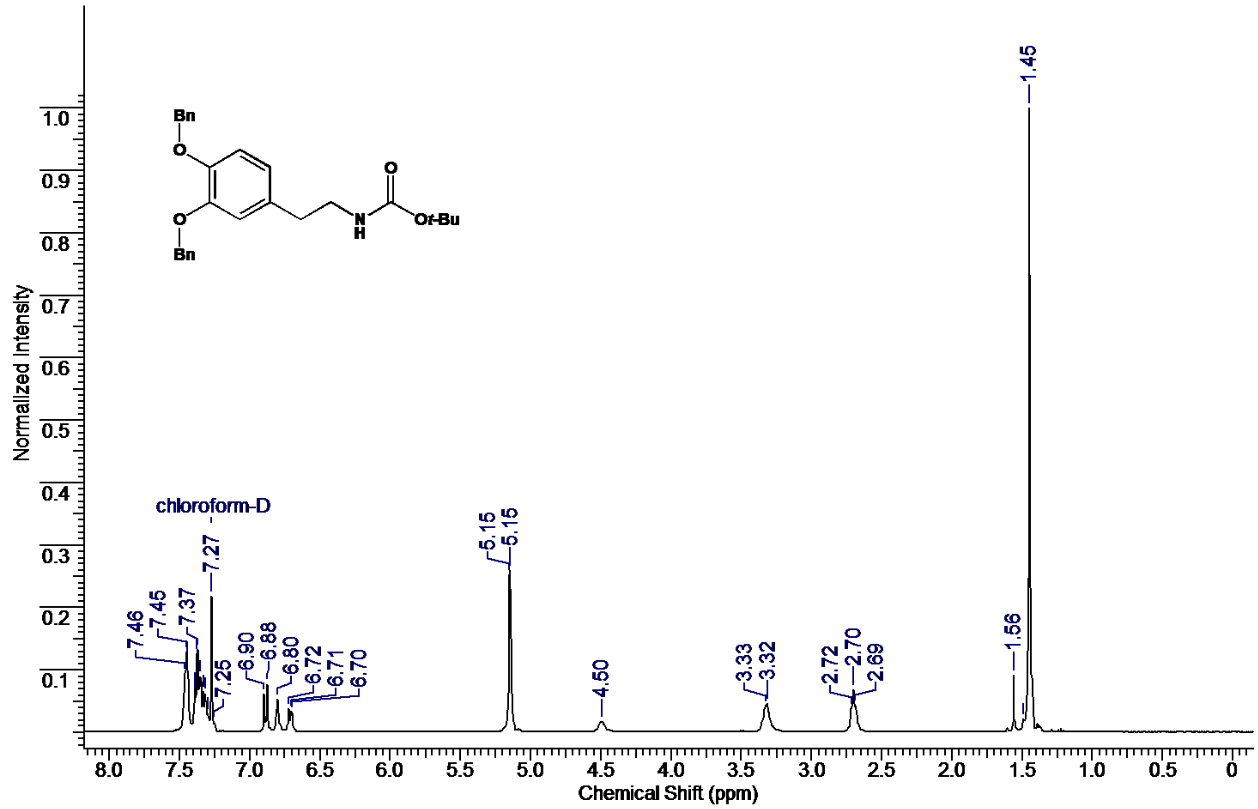

^{1}H NMR of compound 3

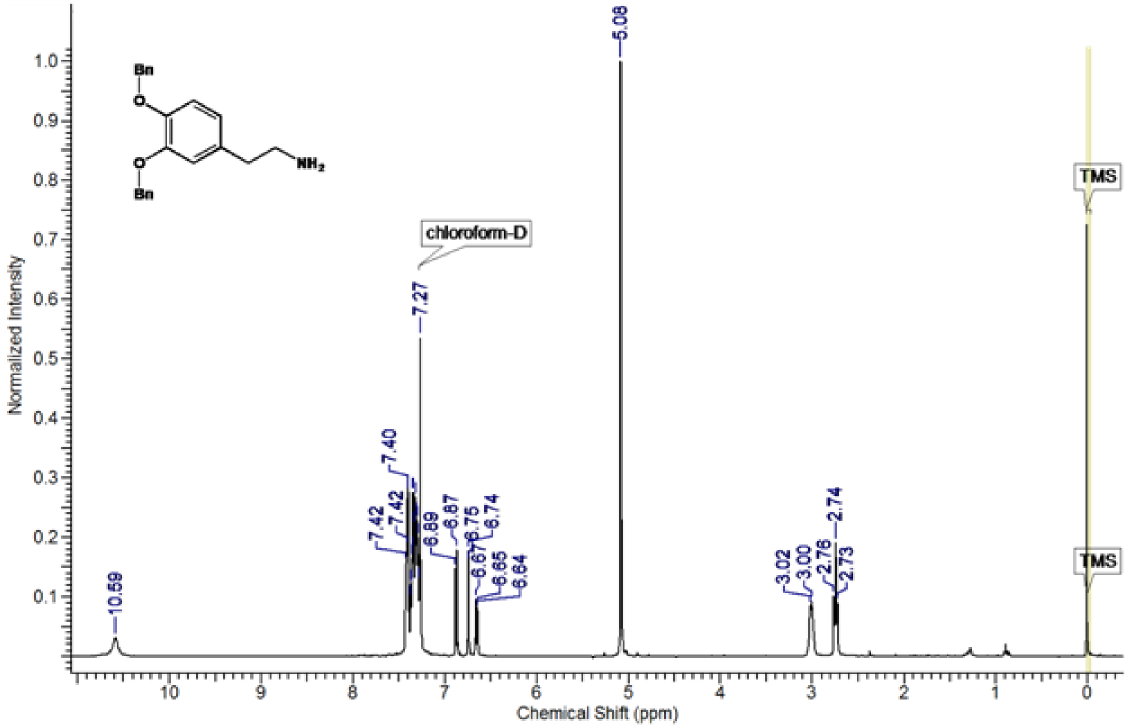

^{1}H NMR of compound 4

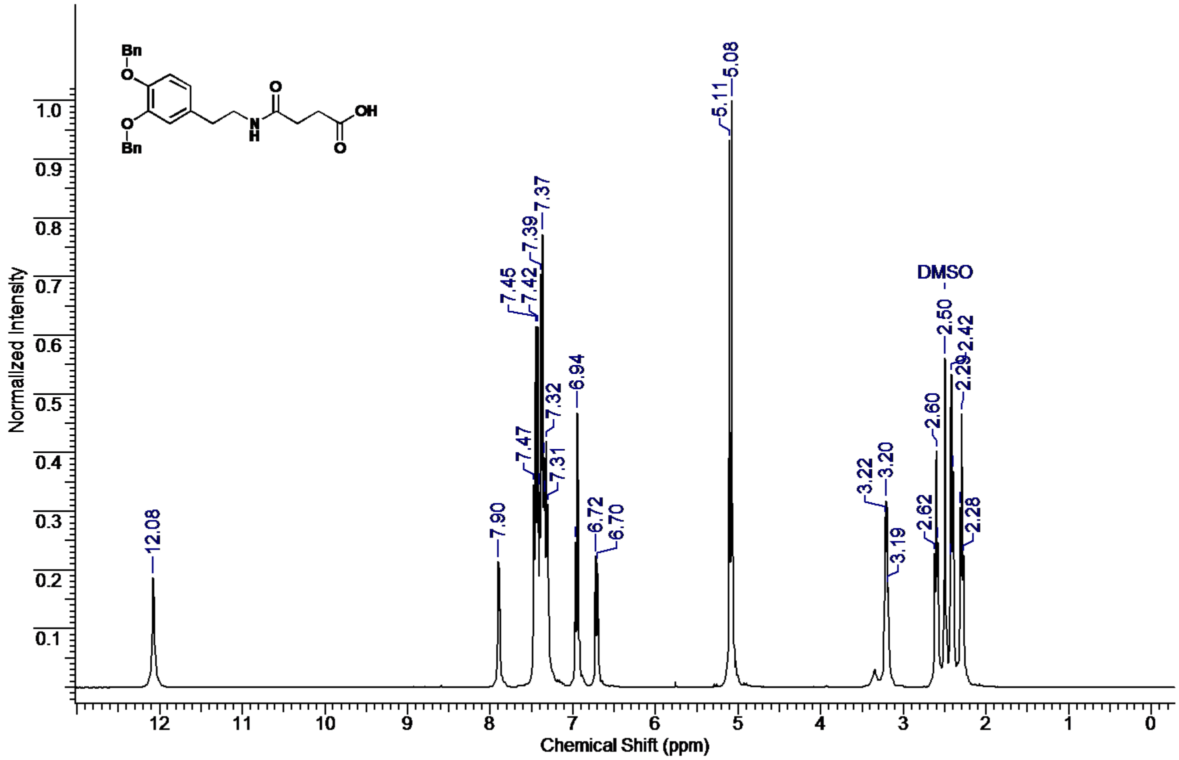

^{1}H NMR of compound 5

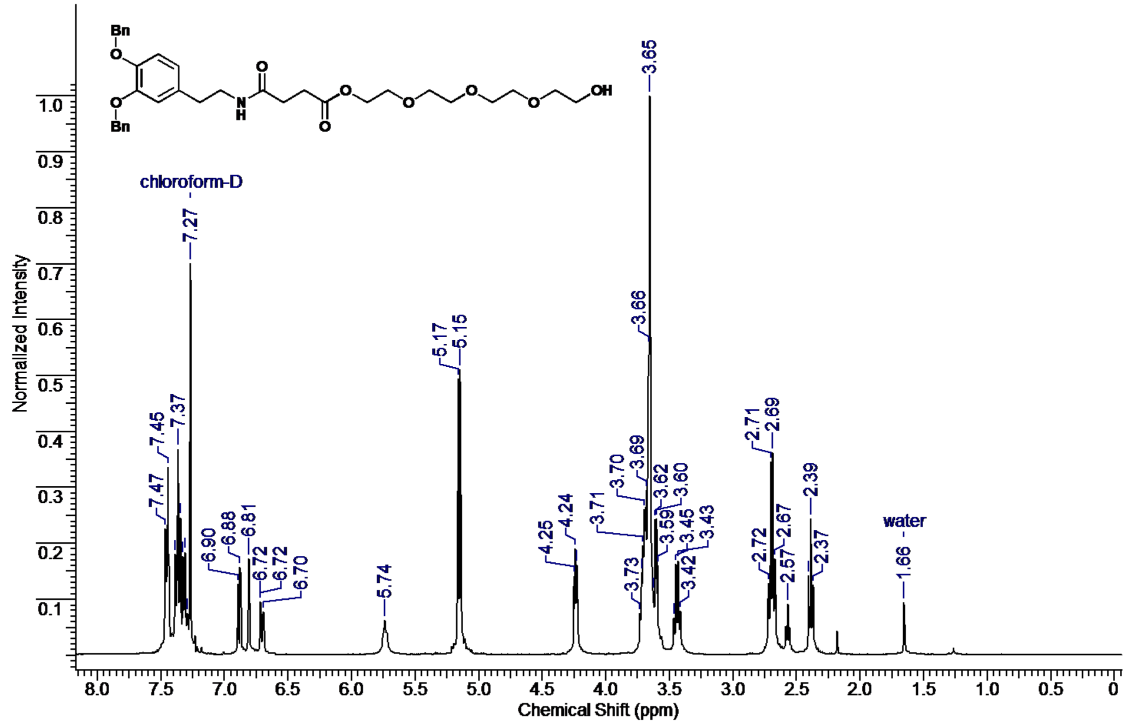

^{1}H NMR of compound 6

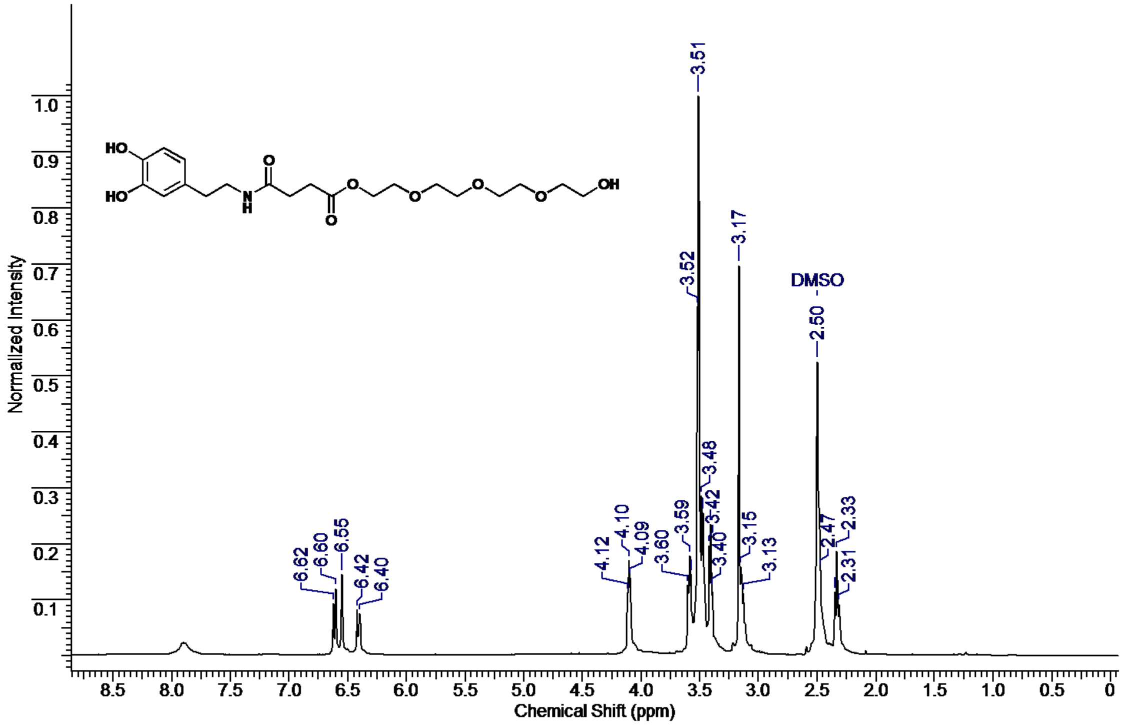

^{1}H NMR of compound 7

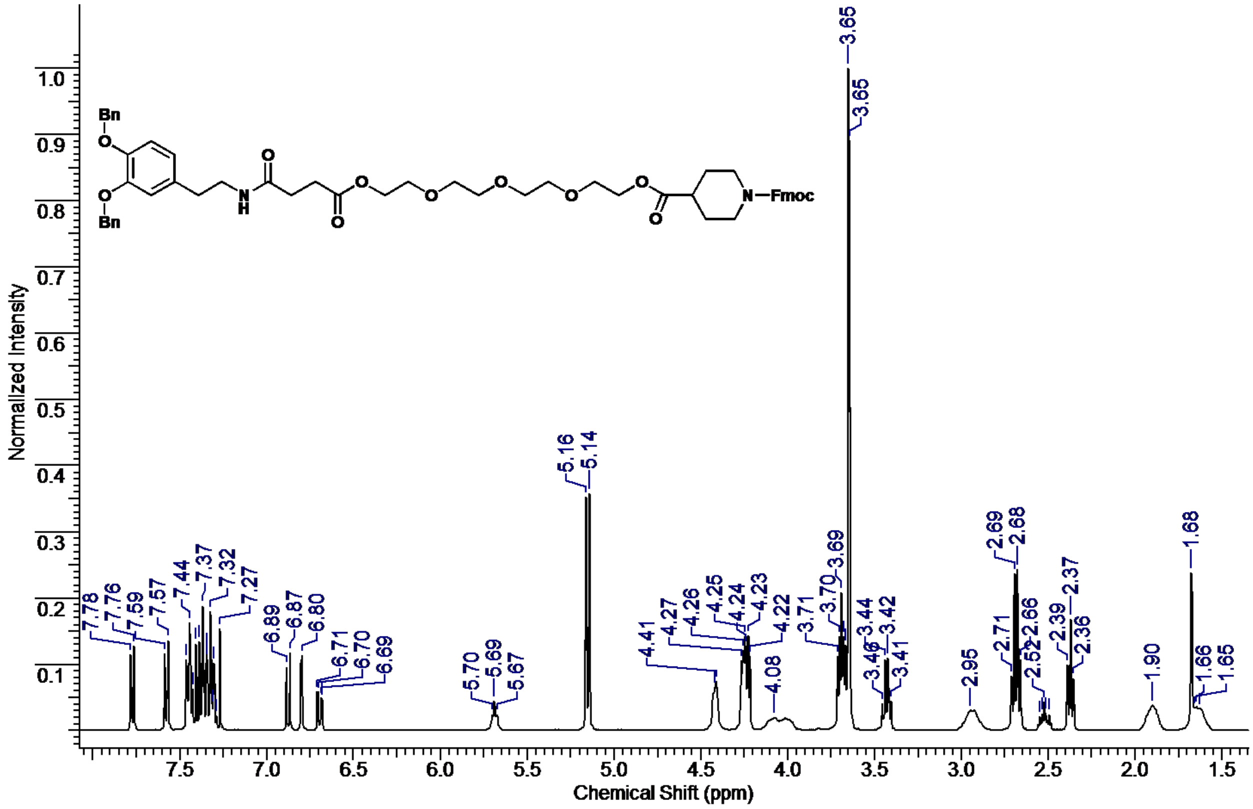

Expansion between 6.5 ppm to 8.0 ppm

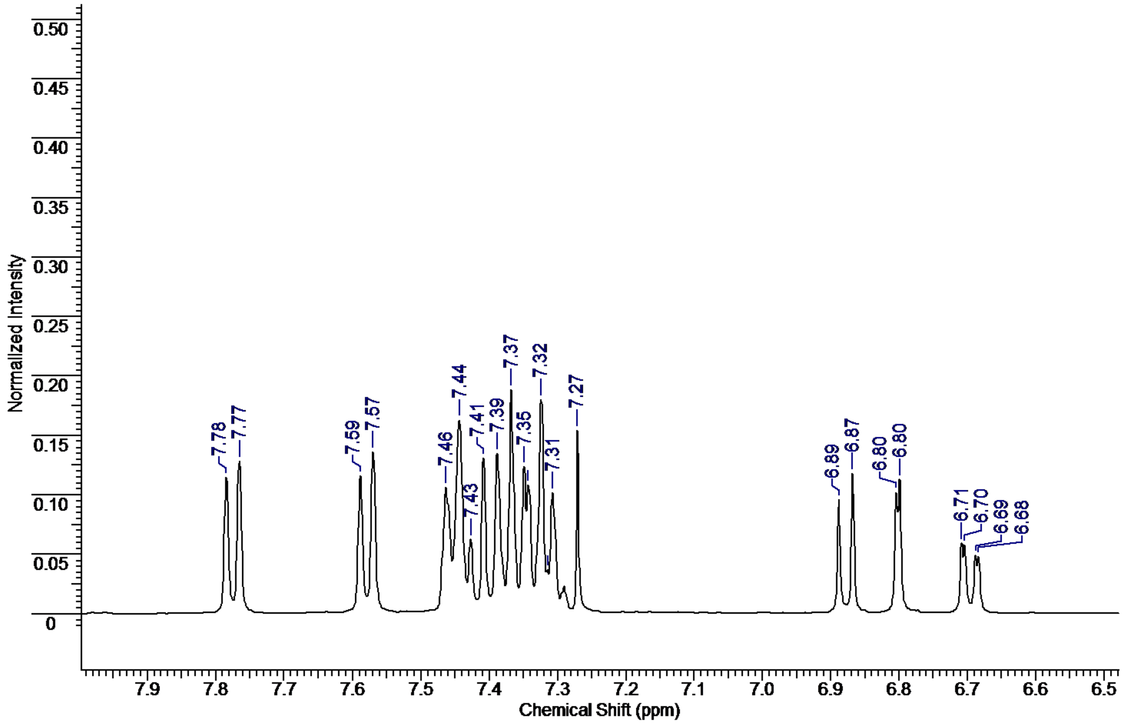

^{1}H NMR of compound 10

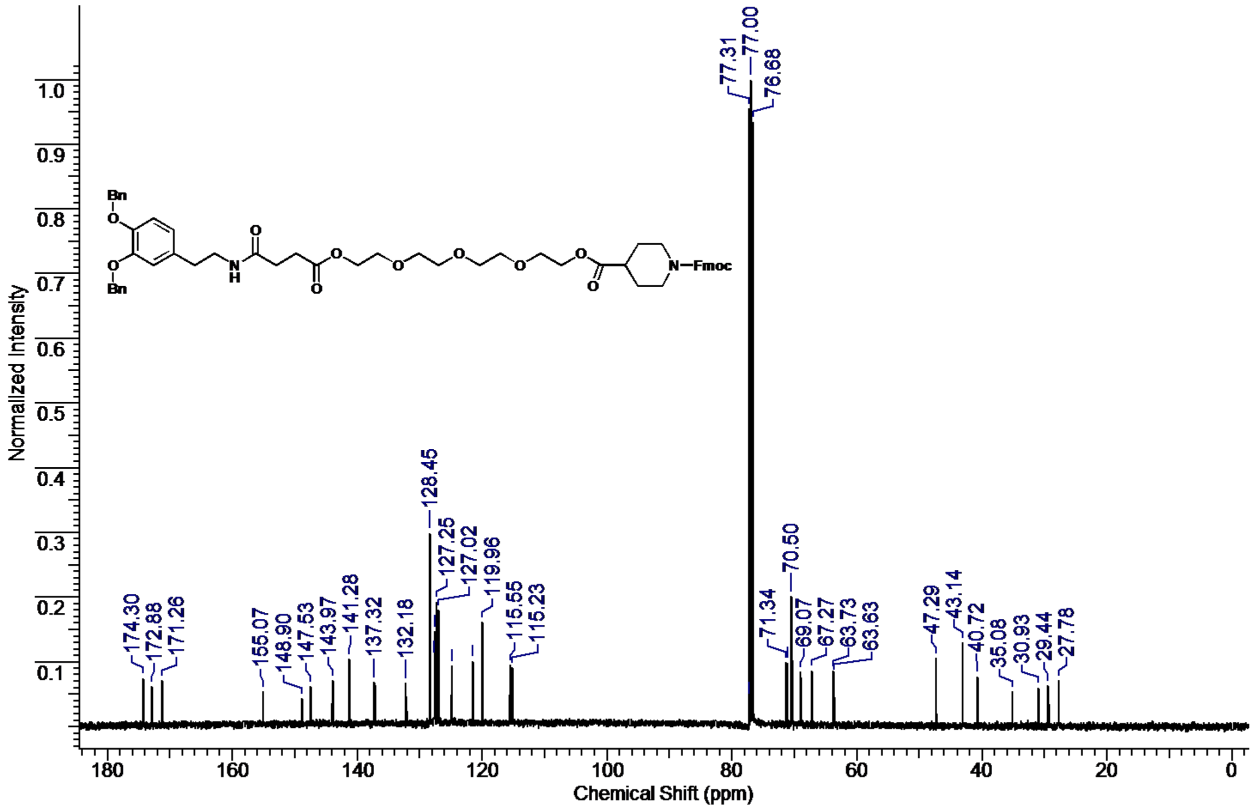

^{13}C NMR of compound 10

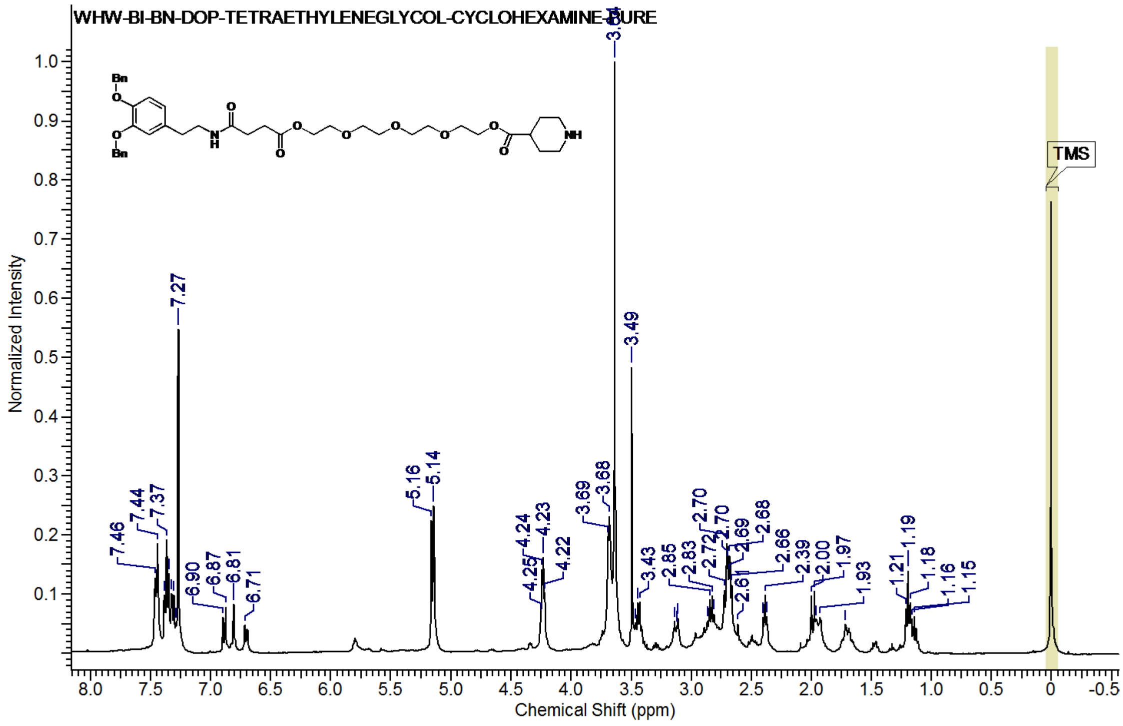

^{1}H NMR of compound 11

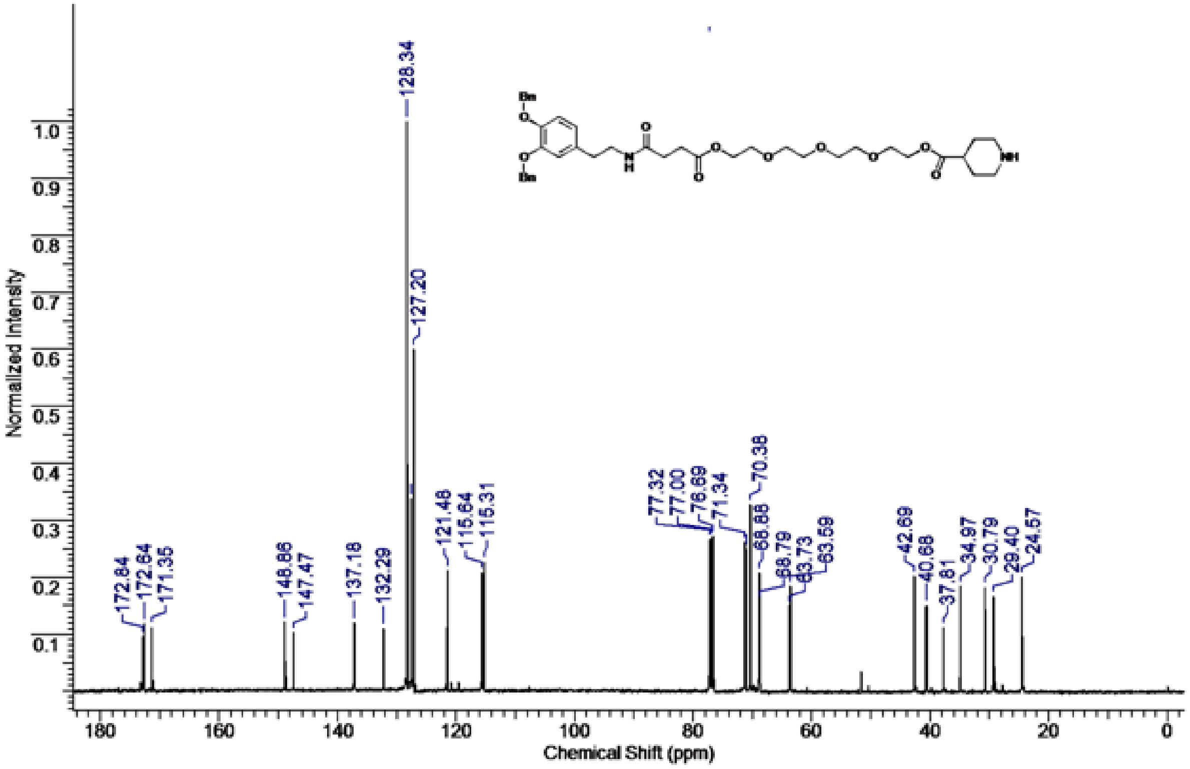

^{13}C NMR of compound 11

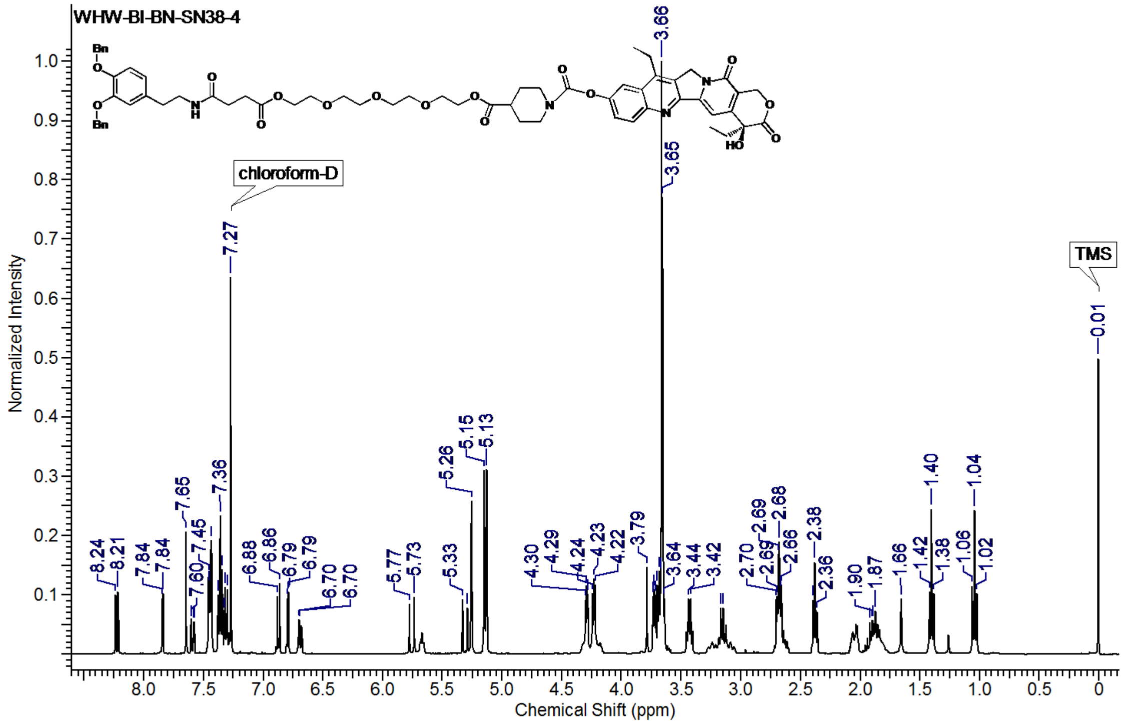

Expansion between 4.7 ppm and 8.4 ppm

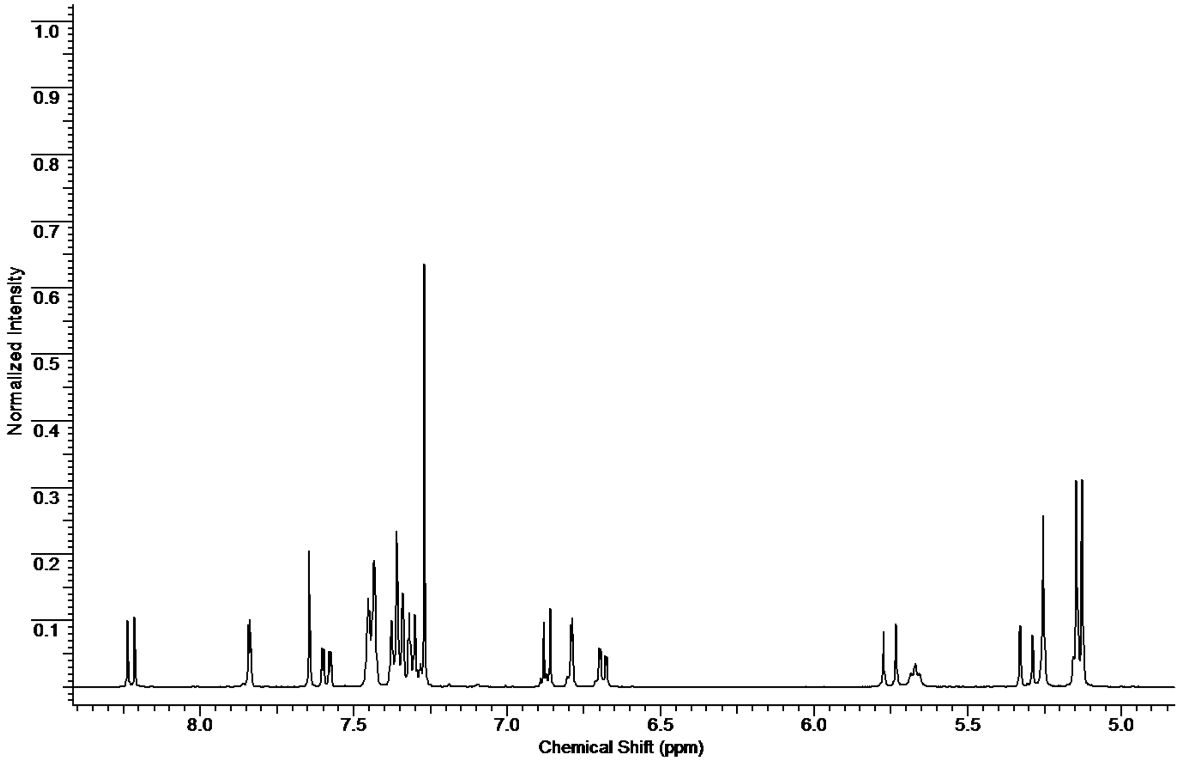

^{1}H NMR of compound 12

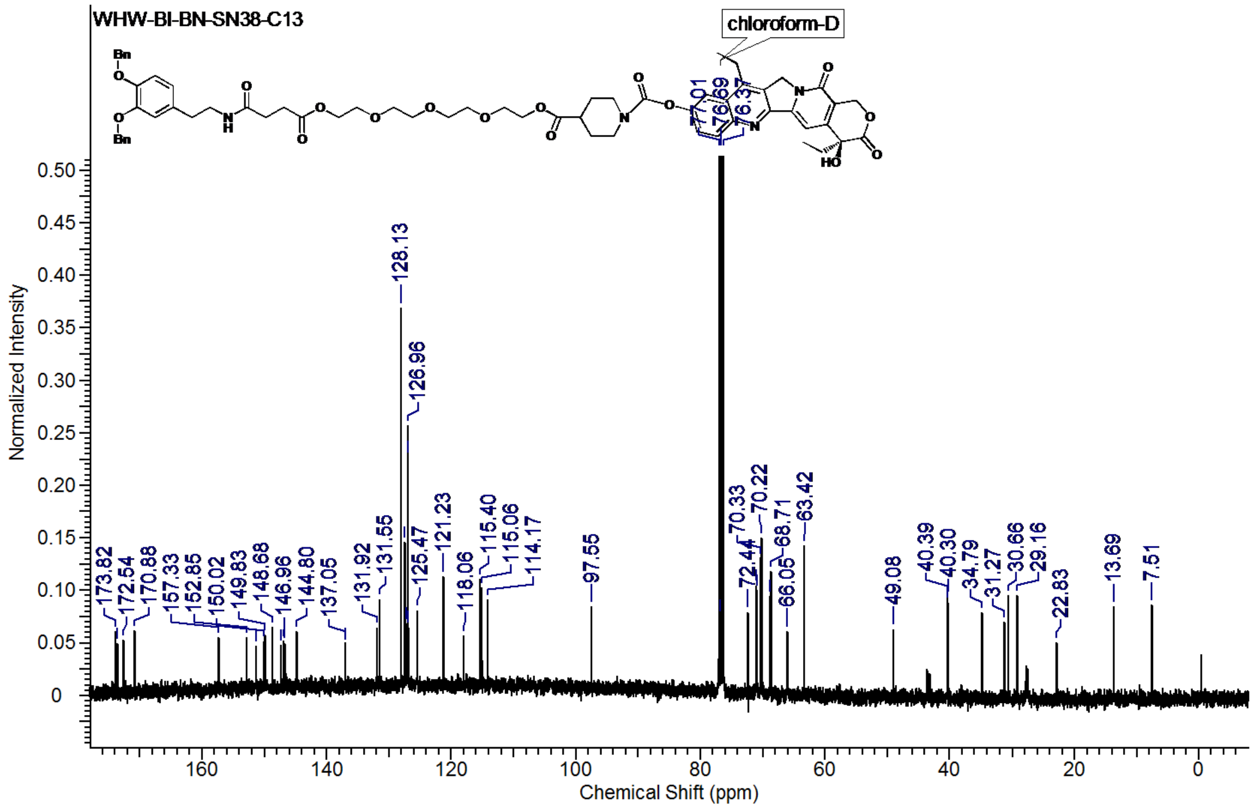

^{13}**C NMR of compound 12**

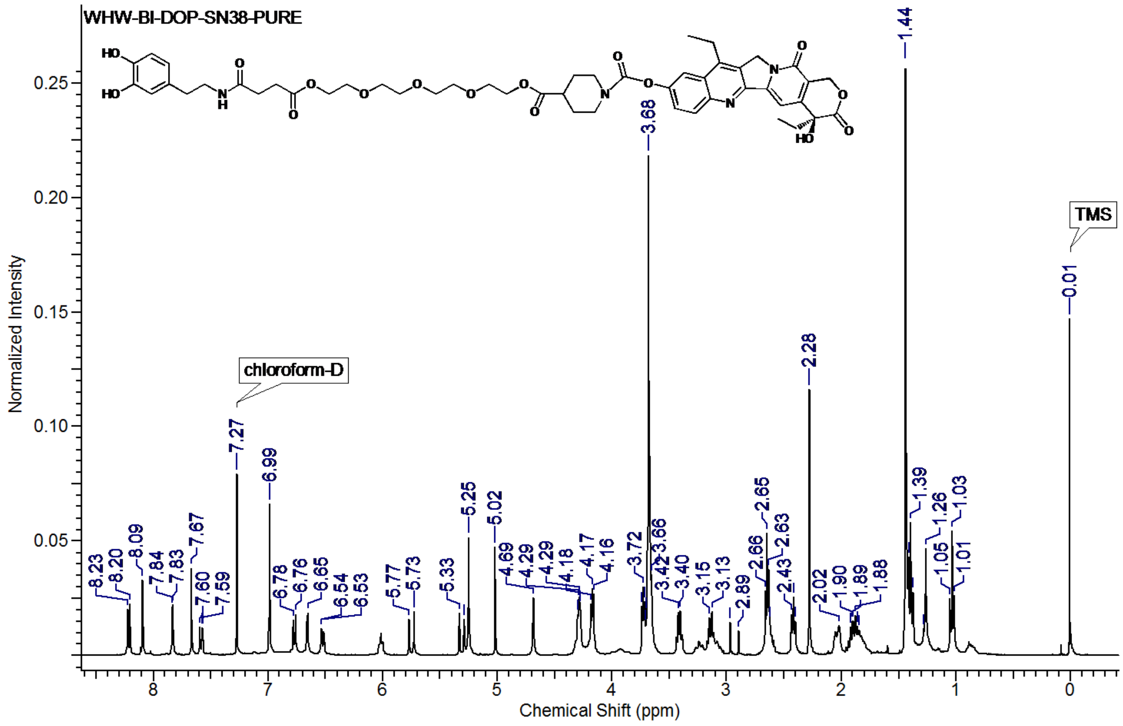

Expansion between 4.7 ppm and 8.5 ppm

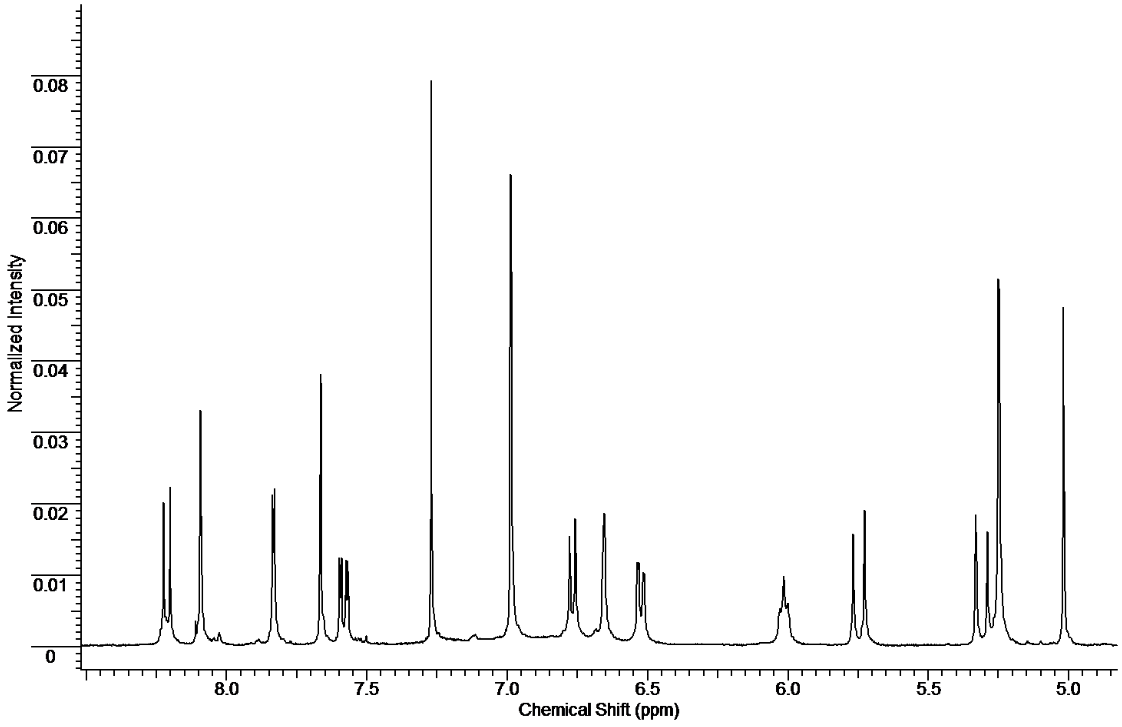

^{1}H NMR of compound 13

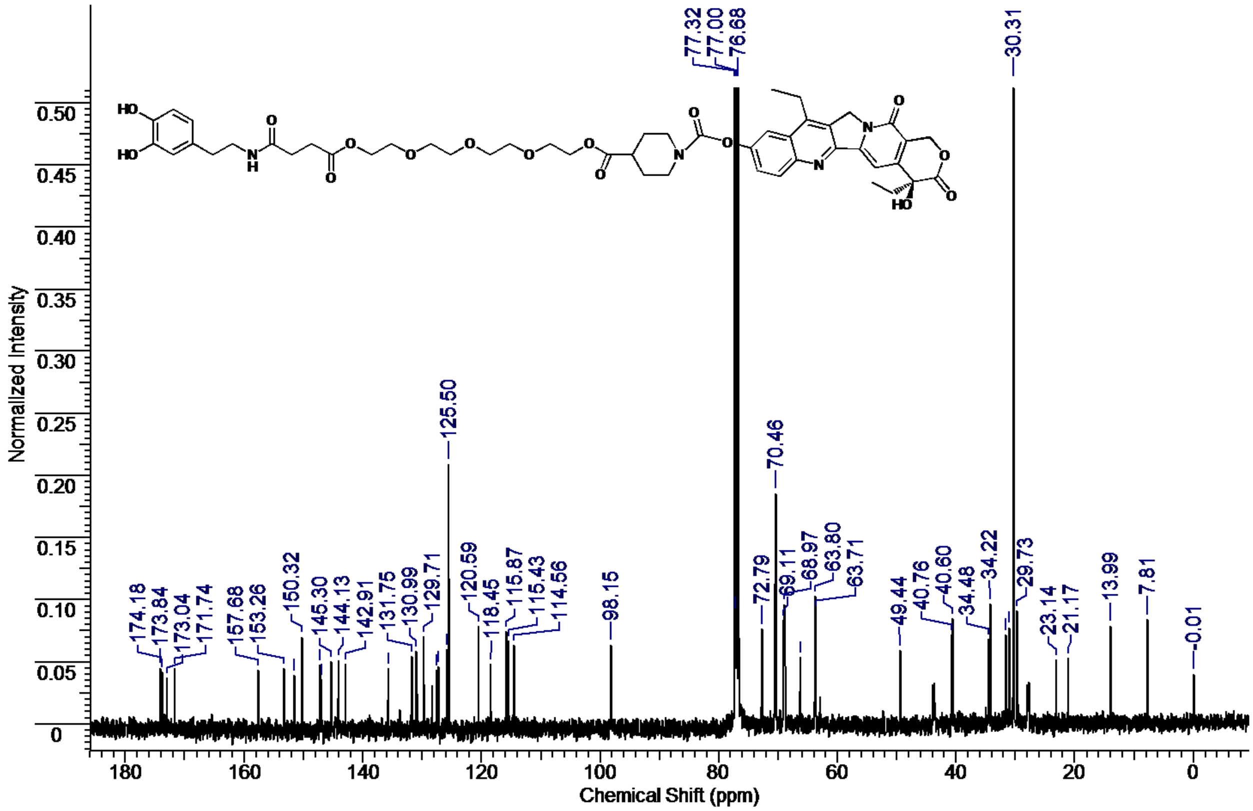

^{13}C NMR of compound 13

MS analysis

MS for compound **10**

Molecular formula = $C_{55}H_{62}N_2O_{12}$

Calculated MS 942.43 Found $(M+Na)^+$ 965.3

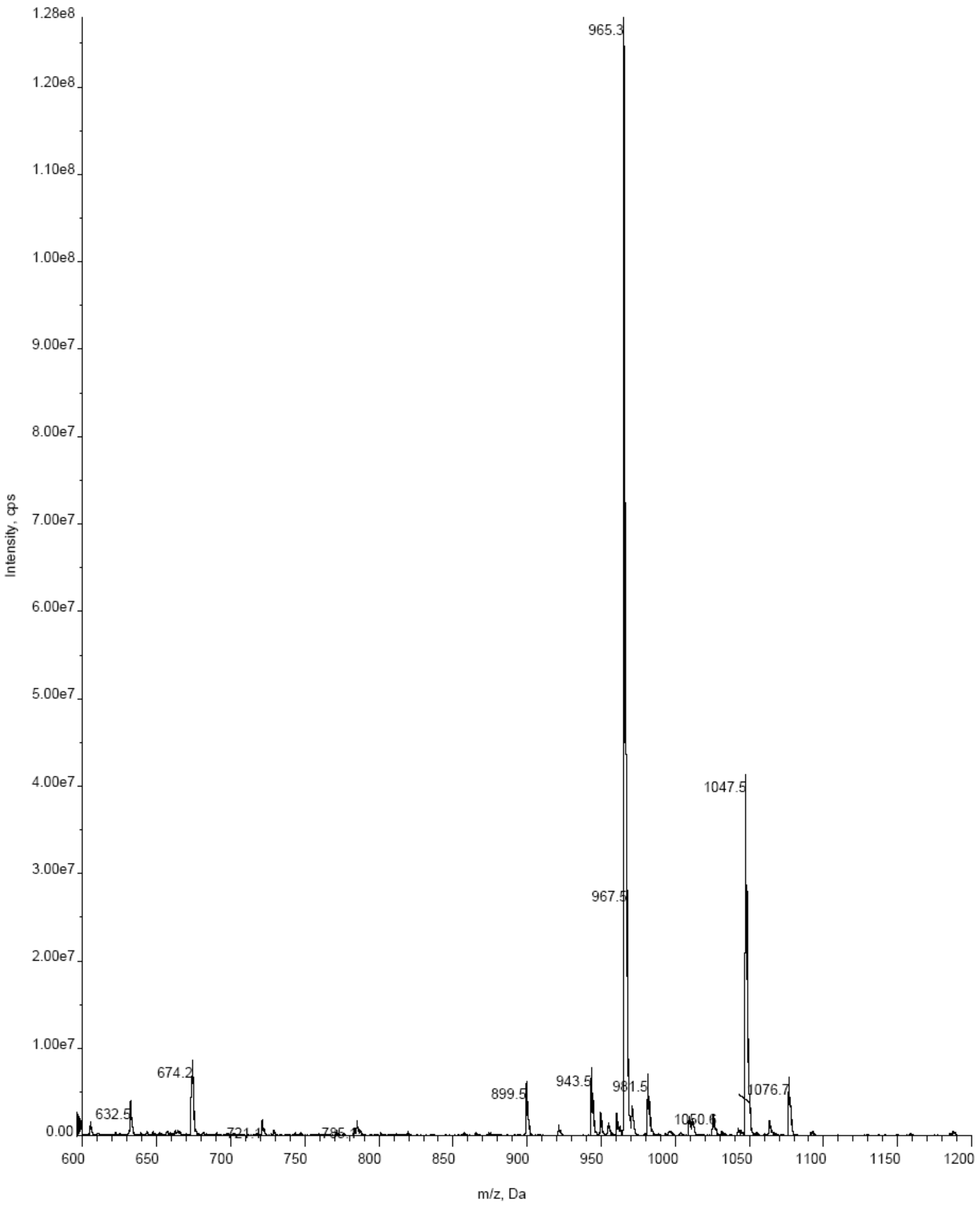

MS for compound **11**

Molecular formula = $C_{40}H_{52}N_2O_{10}$

Calculated MS 720.36 Found $(M+H)^+$ 720.80

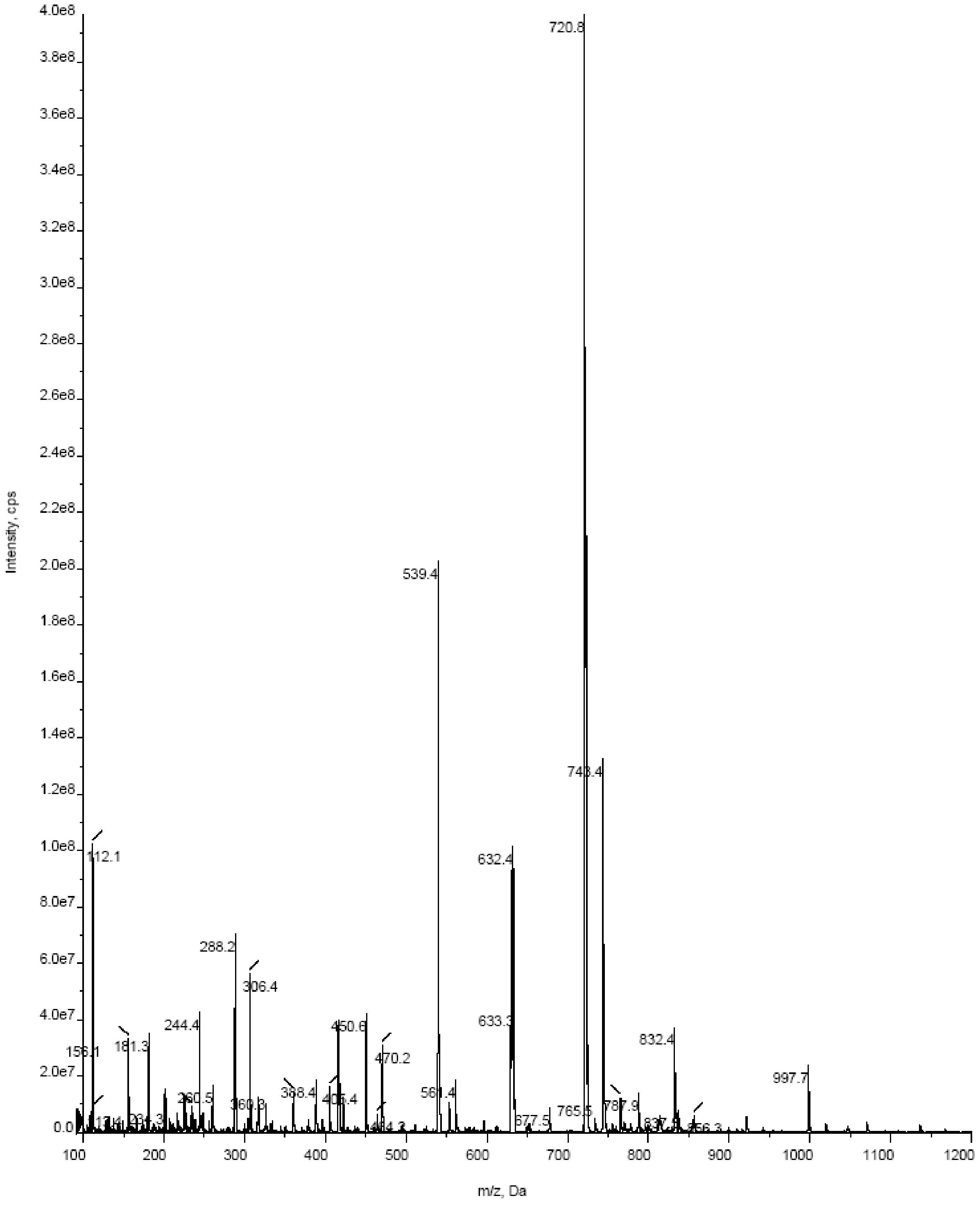

MS for compound **12**

Molecular formula = $C_{63}H_{70}N_4O_{16}$

Calculated MS 1138.48 Found $(M+K)^+$ 1177.50

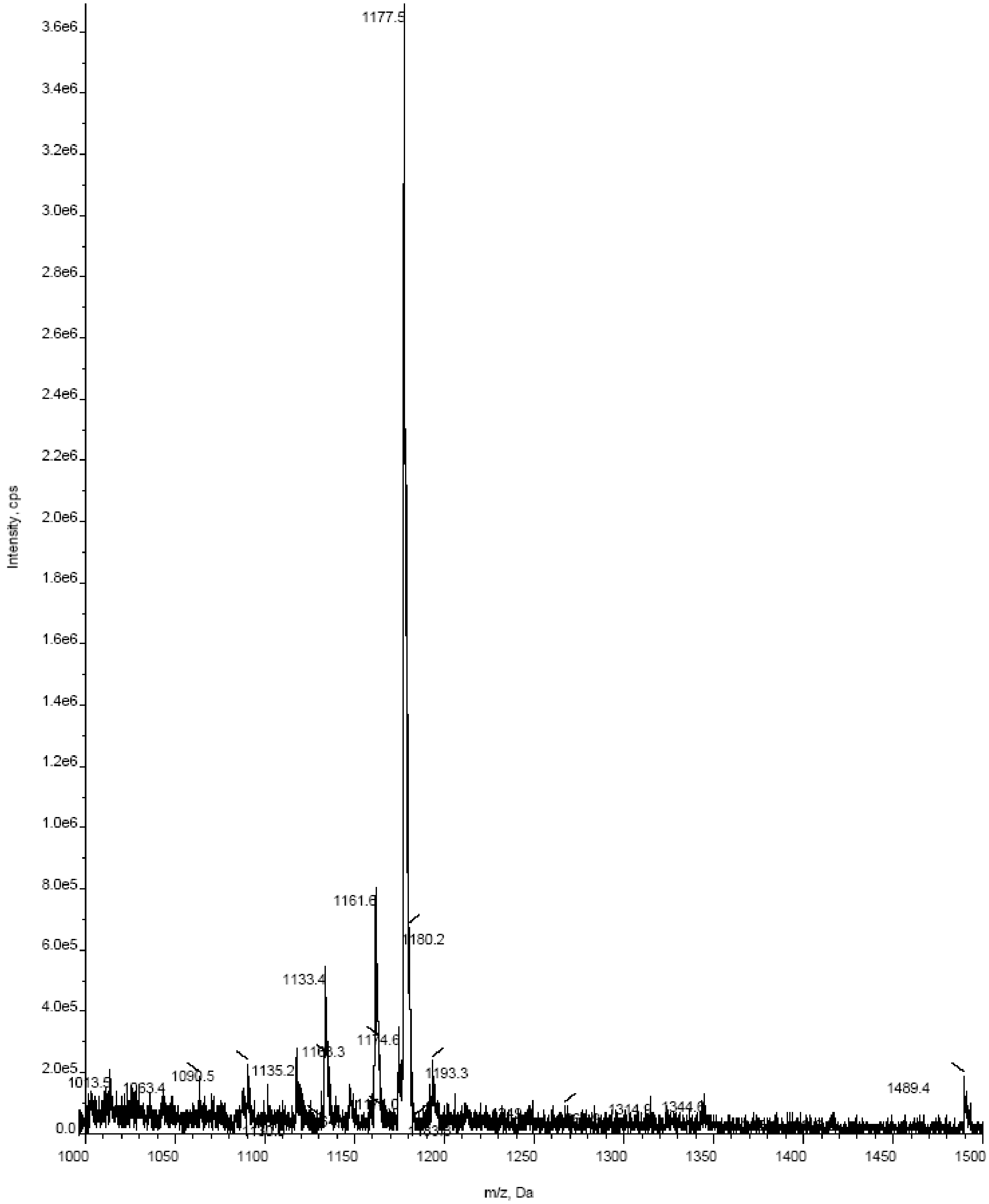

MS for compound **13**

Molecular formula = $C_{49}H_{58}N_4O_{16}$

Calculated MS 958.38 Found $(M+Na)^+$ 981.30

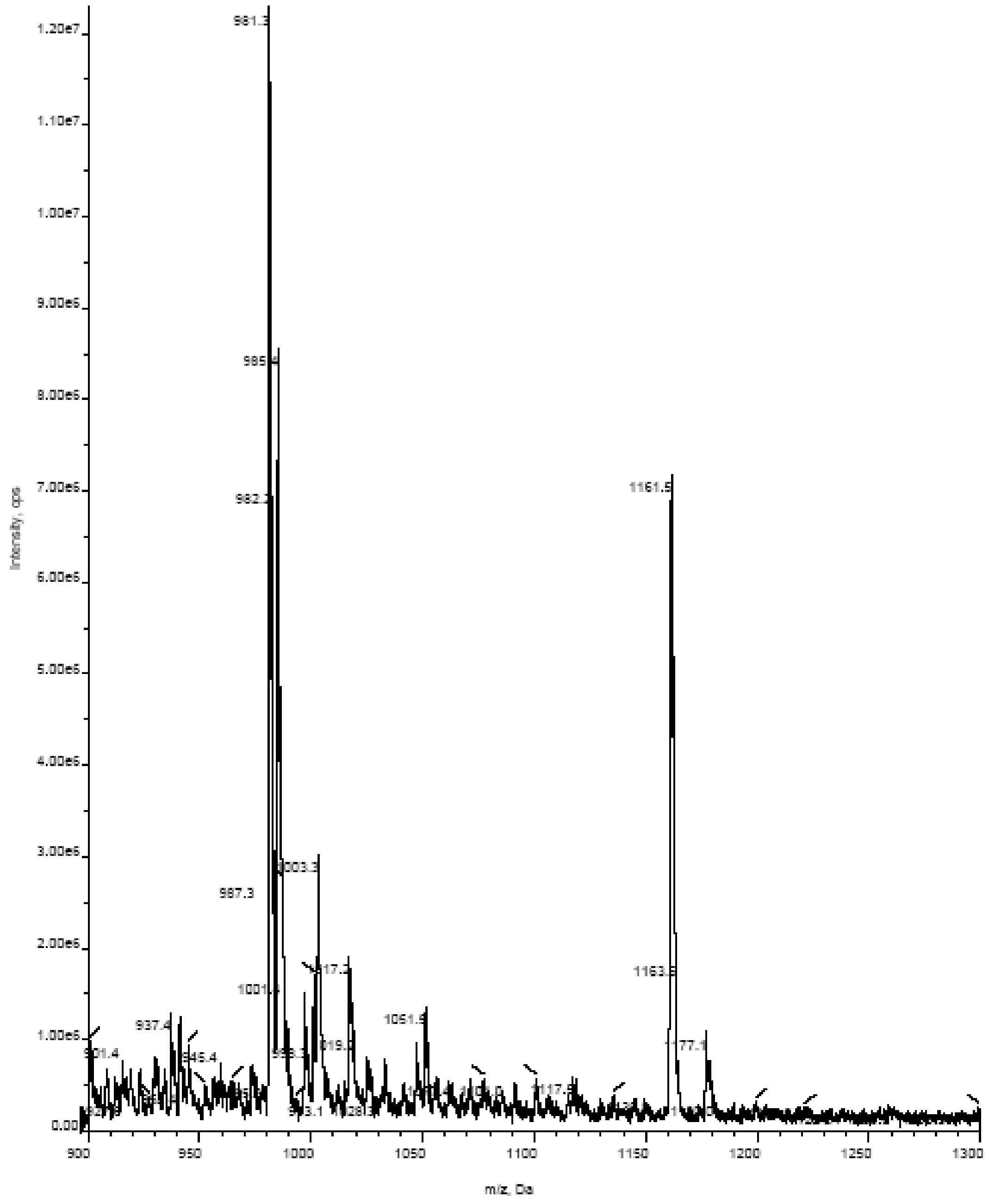

References

1. Lacroix, L. M.; Huls, N. F.; Ho, D.; Sun, X. L.; Cheng, K.; Sun, S. H. *Nano Lett.* **2011,** *11,* 1641. doi:10.1021/nl200110t
2. Xu, C. J.; Xu, K. M.; Gu, H. W.; Zheng, R. K.; Liu, H.; Zhang, X. X.; Guo, Z. H.; Xu, B. *J. Am. Chem. Soc.* **2004,** *126,* 9938. doi:10.1021/ja0464802
3. Fernández, C.; Nieto, O.; Rivas, E.; Montenegro, G.; Fontenla, J. A.; Fernández-Mayoralas, A. *Carbohydr. Res.* **2000,** *327,* 353. doi:10.1016/S0008-6215(00)00073-2
4. Bossmann, S. H.; Ghatlia, N. D.; Ottaviani, M. F.; Turro, C.; Dürr, H.; Turro, N. J. *Synthesis* **1996,** 1313. doi:10.1055/s-1996-4384
